# Therapeutic innovations in triple negative breast cancer: integrating molecular targeting and monoclonal antibody strategies

**DOI:** 10.3389/fonc.2025.1645438

**Published:** 2025-09-22

**Authors:** Prince Ahad Mir, Nishant Kumar, Sukesh K. Gupta, Anureet Kaur, Saeema Farooq, Gurpreet S. Sandhu, Anoop Kumar

**Affiliations:** ^1^ Department of Pharmacognosy and Phytochemistry, Khalsa College of Pharmacy, Amritsar, India; ^2^ Department of Pharmaceutics, Khalsa College of Pharmacy, Amritsar, India; ^3^ Department of Pharmacology, Amity Institute of Pharmacy, Amity University Kolkata, West Bengal, India; ^4^ Department of Pharmacognosy and Phytochemistry, University of Kashmir, Srinagar, Jammu and Kashmir, India; ^5^ Department of Pharmaceutical Analysis, Khalsa College of Pharmacy, Amritsar, India; ^6^ Department of Pharmacy, Dr KN Modi, Institute of Pharmaceutical Education and Research, Modinagar, Uttar Pradesh, India

**Keywords:** TNBC, antitumor, BRCA1 or BRCA2, chemotherapy, signaling pathway

## Abstract

Triple Negative Breast Cancer (TNBC) is a specific kind of breast cancer that is distinguished by the lack of expression of three specific receptors, namely human epidermal growth factor receptor 2 (HER2), estrogen receptor (ER), and progesterone receptor (PR) and are common in women under 40, especially among African American population or those with a BRCA1 genetic mutation. TNBC is characterized by its very aggressive behavior, elevated rates of recurrence, and restricted therapy alternatives in comparison to other subtypes of breast cancer. Chemotherapy is considered the most widely employed therapy against TNBC but experiences off-target toxicity due to its non-selectivity. Such a scenario led to the genetic profiling of the TNBC patients, which led to the identification of several targets and signaling pathways that can be considered as a therapeutic focus for the treatment of TNBC. In this review, we have compiled various therapeutic targets, including androgen receptor (AR) and PI3K/AKT/mTOR, Notch, Wnt/β-catenin, Hedgehog, and TGF-β signaling pathways, which are responsible for the progression of TNBC. In the current therapeutic landscape, the strategic targeting of key signaling pathways, coupled with the development of monoclonal antibody (mAb)-based immunotherapeutic interventions, has emerged as a promising and clinically relevant approach for the management of triple-negative breast cancer (TNBC). The mAbs reduce tumor development, modulate immune responses, and regulate the tumor microenvironment. This review summarizes their mechanisms, signaling pathway targets, clinical applications, and current therapeutic challenges.

## Introduction

Triple-negative breast cancer (TNBC) is a very serious and complex form of breast carcinoma distinguished due to the lack of three prevalent receptors: human epidermal growth factor receptor 2 (HER2/neu), estrogen receptor (ER) and progesterone receptor (PR). The limited expression of receptors in this context hampers the efficacy of hormone-based and targeted treatments that are often used for various forms of breast carcinoma (1). TNBC constitutes around 10-15% of the total breast carcinoma cases, with a higher incidence seen in younger women, especially those of African-American ethnicity. The etiology of TNBC remains incompletely elucidated, while it is postulated to arise from a multifactorial interplay including lifestyle, environmental and genetic determinants (2). Consequently, patients with TNBC have limited treatment options, making it a particularly aggressive and difficult-to-treat form of breast cancer (3).

The prospects of TNBC may exhibit significant variability, contingent upon many variables such as the extent of the tumor, lymphatic participation, and the occurrence of distant metastases. TNBC has a propensity for a heightened incidence of early recurrence and distant metastases in contrast to other variants of breast carcinoma. Additionally, the lack of specific targeted therapies for TNBC makes it particularly challenging to manage and increases the risk of recurrence (4). Early detection and diagnosis of TNBC play a pivotal role in enhancing treatment results. Frequent breast self-examinations, mammographic screenings, and diagnostic breast examinations are necessary for detecting any abnormalities or changes in the breast tissue. If a suspicious lump or mass is detected, a biopsy is performed to confirm the diagnosis and determine the specific subtype of breast cancer (5). TNBC patients may also benefit from genetic screening to discover any inherent genetic alterations, like BRCA1 or BRCA2 alleles linked to a greater possibility of acquiring ovarian and breast cancer. Knowing the genetic profile of the tumor can help guide treatment decisions and identify potential targeted therapies or clinical trials (6).

Despite its aggressive nature, TNBC has shown some responsiveness to immunotherapy, like PD-1 and PD-L1 antagonists. Clinical investigations are under process to assess the potency of immunotherapy in TNBC, both as a standalone treatment and in combination with other therapeutic strategies (7).

In recent times, substantial advances have been made in the domain of targeted therapy, offering the potential for the management of TNBC. Precision medicine is a medical approach that emphasises the customization of therapies according to the specific features of a patient’s tumor. The proposed methodology entails the examination of the genetic and molecular composition of the tumor to discern distinct irregularities that may be effectively addressed via tailored therapeutic interventions (8, 9).

Clinical trials investigating novel treatment approaches, including targeted therapies and immunotherapies, are ongoing for TNBC. Researchers are exploring the potential of targeted agents that inhibit specific signaling pathways involved in TNBC growth and progression (10). Furthermore, researchers are investigating the potential of immunotherapies, including adoptive cell transfer and cancer vaccines, to exploit the immune system’s capabilities in identifying and eradicating TNBC cells (11). In addition to the advancements in treatment approaches, supportive care including strategies to manage the side effects of treatment, alleviate pain, provide psychological care, and immunostimulants plays a crucial role in managing disease and improving the quality of life (12, 13). In addition to therapeutic advances, monoclonal antibodies are expected to become integral components of personalized treatment regimens for TNBC, offering new hope for a historically underserved patient population (14, 15).

## Signaling pathway for triple-negative breast cancer

TNBC lacks the molecular targets commonly used in the treatment of breast carcinoma, like hormone receptors and HER2. The signaling pathways implicated in TNBC are diverse and complex, involving multiple interconnecting networks (16–18). The crucial pathways implicated in TNBC include.

### Phosphoinositide 3-kinase activation

TNBC is frequently associated with aberrant activation of the PI3K pathway. This pathway can be activated by several mechanisms, including (RTKs) receptor tyrosine kinases like IGF-1R (Insulin-like Growth Factor 1 Receptor) and EGFR (Epidermal Growth Factor Receptor) (19). These receptors, when activated by ligands, initiate a signaling cascade that leads to the recruitment and activation of PI3K. The activation of this process is essential in the advancement and growth of many types of malignancies, such as TNBC (20).

**Figure 1:**
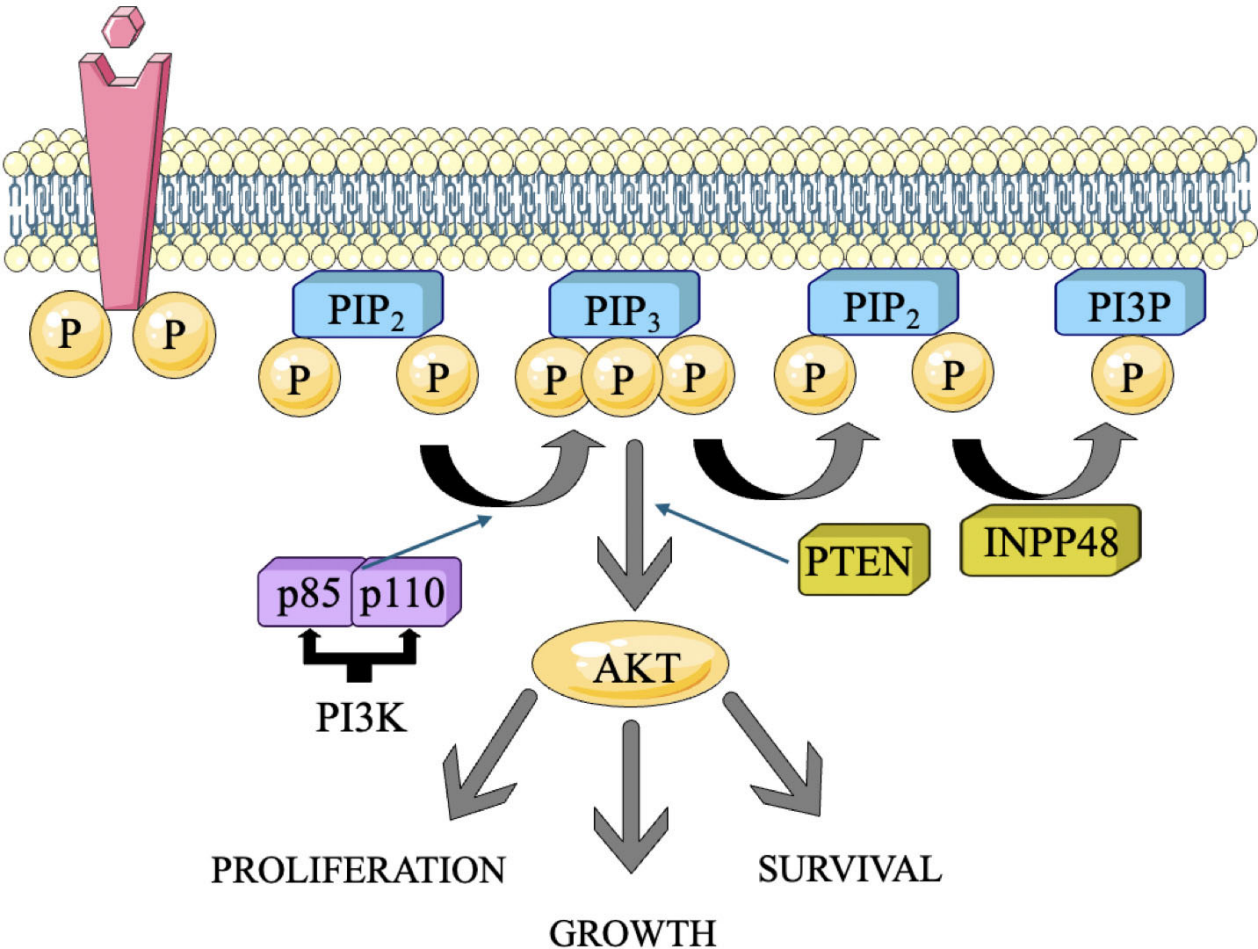
The PI3K pathway and the simplicity of its functions. The primary genomic abnormalities, including mutations and copy number variations (CNVs), identified in each gene in triple-negative breast cancer (TNBC) and their correlation with molecular subtypes (10).

PI3K is a set of kinases that add a phosphate group to the hydroxyl group at the 3rd-position of phosphoinositides. This process converts phosphatidylinositol 4,5-bisphosphate (PIP2) into phosphatidylinositol 3,4,5-trisphosphate (PIP3) (21). Class I PI3Ks are most relevant in cancer, and they exist as heterodimers composed of a catalytic subunit (p110α, p110β, p110δ) and a regulatory subunit (p85α, p85β, p55γ, p55α, p50α) (22) (**Figure 1**). In TNBC, several RTKs are frequently overexpressed or mutated, leading to their constitutive activation. These RTKs, namely IGF-1R and EGFR, may trigger the activation of PI3K by binding to their ligands and beginning subsequent signaling pathways (23). Upon ligand binding to activated RTKs, PI3K is recruited to the cell membrane through interaction between the regulatory subunit and phosphorylated tyrosine residues on the RTK. This localization allows the catalytic subunit of PI3K to phosphorylate PIP2, generating PIP3 (24). PIP3 serves as a second messenger and recruits downstream signaling molecules to the membrane in the form of Akt and PDK1 to the plasma membrane (25, 26).

#### Monoclonal antibodies against PI3K pathway

Monoclonal antibodies that directly activate PI3K in TNBC are not yet prevalent; however, some monoclonal antibodies influence PI3K expression indirectly by targeting immune checkpoints or alternative signaling networks (27). PI3K antagonists, when used in conjunction with these monoclonal antibodies, show potential for the treatment of TNBC by specifically targeting the pathway (28). Furthermore, investigations into innovative monoclonal antibodies that target specific PI3K isoforms represent a promising direction in the treatment of TNBC. Several monoclonal antibodies pertinent to TNBC and the phosphoinositide 3-kinase (PI3K) pathway are identified (29).

♣ Herceptin (Trastuzumab) a monoclonal antibody used in the treatment of HER2-positive breast cancer. It targets the HER2 receptor, inhibiting tumor growth and promoting apoptosis in cancer cells. Its efficacy has been established through numerous clinical trials, demonstrating improved outcomes in patients with this specific cancer subtype (30). Trastuzumab, that does not specifically target PI3K however, influences the PI3K/Akt cascade through downstream effects by inhibiting HER2 essential for triggering PI3K pathway (31). This treatment is primarily indicated for HER2-positive breast cancer; however, certain studies have investigated its potential application in TNBC, particularly in the HER2-negative subset that may exhibit significant signaling alterations (32).

♣ Margetuximab a monoclonal antibody targeting HER2, analogous to trastuzumab. It has demonstrated potential to influence PI3K signaling circuits (33). Preclinical findings indicate that margetuximab elicits a superior immune response relative to trastuzumab, potentially influencing PI3K-related downstream signaling in certain HER2-positive and even TNBC cells exhibiting HER2 overexpression (34).

♣ Anti-PD-L1 antibodies, such as Atezolizumab and Durvalumab, are utilized in therapeutic applications. While these antibodies do not directly activate the PI3K pathway, they regulate the immune reaction, and stimulating T cells that can indirectly affect PI3K signaling pathways (35). Immune checkpoint inhibitors are being utilized more frequently in TNBC, especially in conjunction with other treatments, including chemotherapy or targeted PI3K inhibitors (36).

♣ Bavituximab is a monoclonal antibody that targets phosphatidylserine, a molecule associated with the activation of PI3K/Akt signaling pathways. While this does not directly activate PI3K, it can indirectly affect the pathway, resulting in enhanced immunity against tumors and apoptosis in cancer cells (37).

♣ CureD3: Targeted PI3K isoforms. Monoclonal antibodies targeting specific isoforms of PI3K have been investigated in preclinical models that block the irregular PI3K pathway in cancers, like TNBC (29).

### Protein kinase B activation

**Figure 2:**
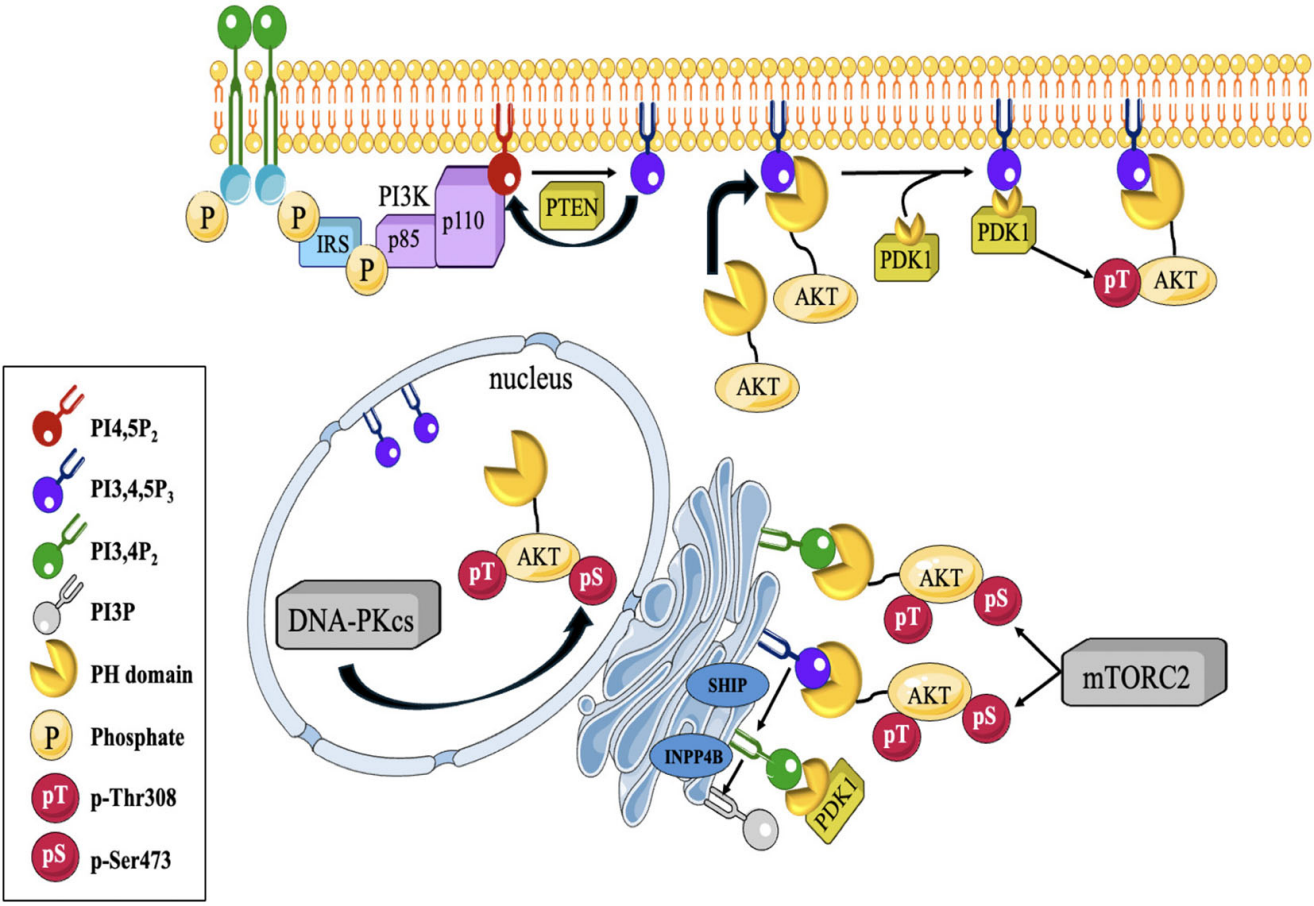
Depicts a diagrammatic illustration of compartmentalized Akt activation. PI(3,4,5)P3 synthesis at the cellular membrane is contingent upon the activation of PI3K by active RTKs and GPCRs downstream. Afterwards, Akt is brought to the cell membrane by attaching its PH- domain to the produced (PI(3,4,5)P3. Akt binds to PI (3,4,5)P3, which enables phosphorylation at T308 by PDK1, a PH-domain containing protein, and at S473 by mTORC2 or DNA-PKCS at different locations within the cell. Akt may also be triggered at endomembranes by attaching to P13,4P2, which is produced via a process catalyzed by SHIP from P1(3,4,5)P3. PTEN and INPP4B restrict Akt activation by regulating the levels of PI(3,4)P2 (42).

AKT, plays a pivotal part in the development and advancement of several types of malignancies, such as TNBC (38). It is a serine/threonine protein kinase that participates in several physiological functions, like cell survival, metabolism, growth, angiogenesis and proliferation. It operates after several signaling pathways, such as the PI3K/AKT pathway, which is often disrupted in TNBC (39). Activation of AKT in TNBC can occur through several mechanisms. One of the most common mechanisms is the overactivation of the PI3K pathway. Once activated, AKT phosphorylates and modulates the activity of numerous downstream effectors involved in cell survival and proliferation (40, 41) (**Figure 2**). The dysregulation of PI3K/AKT pathway can occur through various mechanisms, including genetic alterations, such as mutations in the PI3K catalytic subunit (PIK3CA) or loss of the tumor suppressor gene PTEN, which negatively regulates the pathway. Additionally, other signaling molecules, such as receptor tyrosine kinases (RTKs) or growth factor receptors, can activate AKT through the PI3K pathway (43).

#### Monoclonal antibodies against AKT pathway

Akt, serves as a crucial regulator of numerous cellular processes, including survival, growth, proliferation, and metabolism. The Akt signaling pathway is often impaired in various cancers, such as TNBC, which contributes to chemoresistance, tumor progression, and unfavorable prognosis (42). Monoclonal antibodies (mAbs) that specifically activate Akt in TNBC are infrequent and not extensively investigated in contemporary treatments. Indirect stimulation or modulation of the Akt pathway represents a more common strategy in cancer therapy (44). Monoclonal antibodies may affect the Akt pathway via downstream mechanisms, either by inhibiting alternative signaling pathways (such as HER2 or immune checkpoint pathways) or by interfering with tumor dynamics (45).

♣ Anti-PD-L1 Antibodies: Atezolizumab (Tecentriq) and Durvalumab (Imfinzi) PD-L1 antibodies inhibit immune checkpoint signaling, thereby augmenting T-cell-mediated immune responses (46). Activated immune cells can influence tumor signaling pathways, such as Akt. PD-L1 inhibitors do not directly trigger Akt; however, they may enhance immune system activation, resulting in Akt suppression or indirectly influencing Akt in tumor cells (47).

♣ Antibodies targeting the Anti-Insulin-Like Growth Factor Receptor (IGF-1R): IGF-1R is a receptor that, upon activation, initiates multiple downstream signaling pathways, notably the PI3K/Akt pathway (48). Inhibition of IGF-1R signaling through monoclonal antibodies such as figitumumab (CP-751,871) may impede aberrant Akt activation in tumor cells, thereby potentially diminishing tumor growth. Monoclonal antibodies that target IGF-1R may indirectly influence Akt signaling by inhibiting tumor survival signals (48, 49).

♣ Anti-VEGF (Vascular Endothelial Growth Factor) agents Immunoglobulins: Bevacizumab (Avastin) inhibits the VEGF pathway, which plays a critical role in angiogenesis, the process of new blood vessel formation (50). This indirectly influence Akt activation in both tumor cells and endothelial cells (51). Bevacizumab has been utilized alongside chemotherapy for the treatment of TNBC. By inhibiting vascular endothelial growth factor (VEGF), it may obstruct certain survival signals that would typically activate Akt within the tumor microenvironment (52).

♣ Antibodies targeting Interleukin-6 (IL-6): Tocilizumab, also known as Actemra, is a monoclonal antibody that inhibits interleukin-6 (IL-6) receptors, utilized primarily in the treatment of autoimmune diseases such as rheumatoid arthritis and systemic juvenile idiopathic arthritis (53). Interleukin-6 (IL-6) is a cytokine that plays a role in inflammation and tumor progression and has the capacity to activate multiple signaling pathways, such as Akt (54). Tocilizumab indirectly modulate Akt signaling by blocking IL-6 receptor signaling and inhibiting the inflammatory microenvironment, which plays a crucial role in cancer progression (55).

♣ Combination therapies Numerous monoclonal antibodies are currently under investigation in conjunction with PI3K inhibitors or mTOR inhibitors to enhance targeting of the Akt pathway in TNBC (56). These combinations may indirectly modulate Akt signaling, leading to tumor cell apoptosis and diminished proliferation (57). Alpelisib (BYL719) a selective inhibitor of PI3Kα that, in conjunction with specific monoclonal antibodies (e.g., trastuzumab or immune checkpoint inhibitors), has the potential to diminish Akt activation in TNBC (58).

### Mammalian target of rapamycin activation

**Figure 3:**
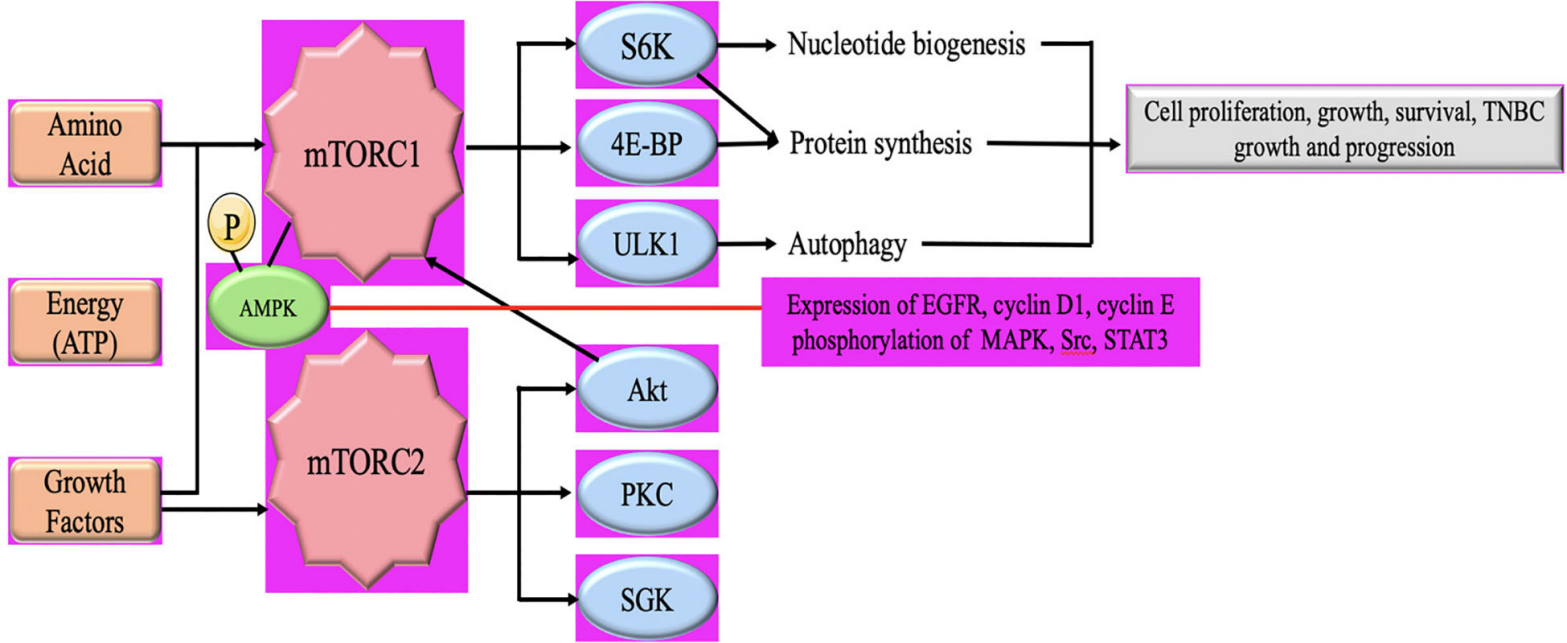
Schematic illustration of the AMPL/mTOR signaling pathway in TNBC Tumor growth and progression. mTORC1 comprises mTOR, mammalian lethal with sec-13 protein 8 (mLST8), and regulatory-associated protein of mammalian target of rapamycin (RAPTOR). mTORC1 is activated by growth factors, nutrients (amino acids), and cellular energy. mTORC2 consists of mTOR, mLST8, mammalian stress activated map kinase-interacting protein 1 (mSIN1) and rapamycin-insensitive companion of mTOR (RICTOR) and is activated by growth factors (62).

mTOR is a protein kinase that plays a crucial role in cell growth, metabolism, and survival. It is a central regulator of various cellular processes, including protein synthesis, autophagy, and cell proliferation (38). Dysregulation of mTOR signaling has been implicated in the development and progression of several types of cancer, including TNBC. In TNBC, mTOR activation has been observed in a significant proportion of cases. Hyperactivation of mTOR signaling may arise from many pathways, such as the inactivation of adverse regulators like PTEN, and the stimulation of beneficial regulators like AKT and PI3K (59, 60). These modifications result in heightened mTOR activity, which then triggers the activation of the following pathways responsible for cell survival and development. mTOR signaling promotes cell cycle progression by regulating the translation of key proteins involved in cell growth and division. Enhanced mTOR activity in TNBC can contribute to uncontrolled cell proliferation, leading to tumor growth (61) (**Figure 3** **).** This activation can further promote cell development by suppressing apoptosis and also plays a pivotal role in the development of resistance to chemotherapy and targeted therapies in TNBC. Inhibiting mTOR signaling may sensitize TNBC cells to chemotherapy and enhance treatment response (63, 64). mTOR also regulates cellular metabolism, including glucose and lipid metabolism. In TNBC, mTOR activation can promote the uptake and utilization of nutrients, providing energy and resources for tumor growth (65).

#### Monoclonal antibodies against mTOR pathway

The mTOR pathway comprises two complexes, mTORC1 and mTORC2, whose activity is meticulously regulated by several cellular signals, including growth hormones, nutrition, and stress indicators. Dysregulation of mTOR signaling has a significant role in carcinogenesis, making it a crucial therapeutic target, especially in TNBC (62). Currently, no monoclonal antibodies are authorized to directly activate mTOR in TNBC; however, some monoclonal antibodies influence mTOR signaling indirectly by targeting upstream pathways such as HER2, VEGF, PD-L1, and IGF-1R (66). These medicines can regulate mTOR activity, resulting in the suppression of tumor development. Nonetheless, mTOR inhibitors and combination treatments using monoclonal antibodies that target alternative pathways have shown potential in the treatment of TNBC via the modulation of mTOR signaling (67).

♣ Monoclonal Antibodies that Indirectly Modulate mTOR in TNBC:

Anti-PD-L1 Antibodies (e.g., Atezolizumab, Durvalumab): PD-L1 participates in immune evasion mechanisms, and the inhibition of PD-L1 with monoclonal antibodies such as atezolizumab (Tecentriq) and durvalumab (Imfinzi) may augment T-cell activation (68). Immune activation, including the stimulation of T-cell receptors (TCRs), may initiate many downstream signaling cascades, notably the PI3K/Akt/mTOR pathway (69). Although PD-L1 monoclonal antibodies are not intended to activate mTOR, they may indirectly augment immune responses that influence mTOR signaling inside the tumor microenvironment (70).

♣ Anti-IGF-1R antibodies: IGF-1R participates in the activation of the PI3K/Akt/mTOR signaling pathway. Monoclonal antibodies, such as figitumumab, inhibit the signaling pathway that results in mTOR activation. These monoclonal antibodies inhibit its aberrant activation, resulting in decreased cell survival and proliferation (71).

♣ Anti-VEGF antibodies: Inhibiting VEGF signaling using monoclonal antibodies such as bevacizumab (Avastin) might diminish the activation of downstream pathways, including the PI3K/Akt/mTOR pathway, since angiogenesis and tumor viability depend on these signaling mechanisms. Bevacizumab indirectly decreases mTOR by its impact on the tumor microenvironment (72).

### Nuclear factor-κB activation

**Figure 4:**
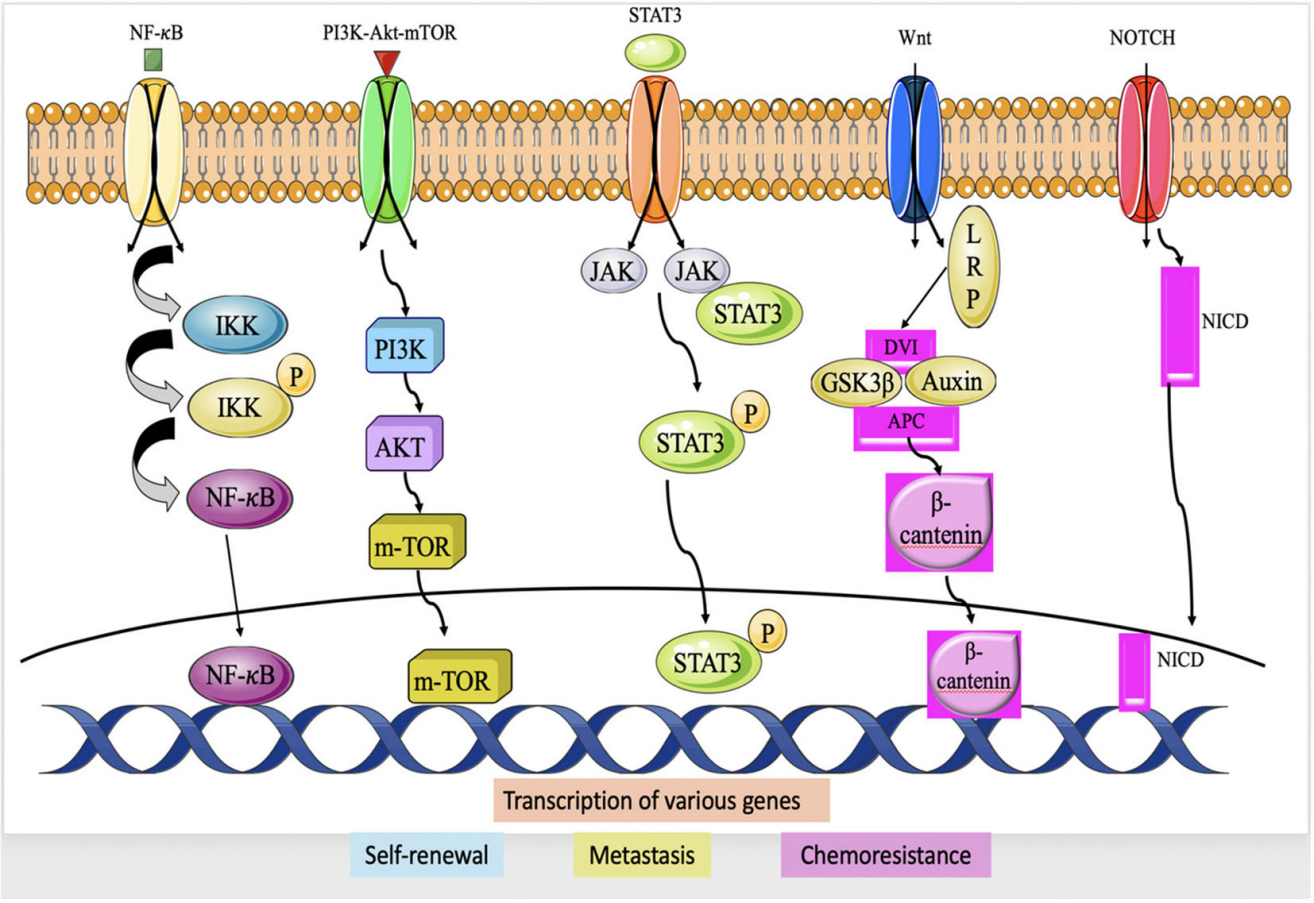
Major Signaling pathway in TNBC chemoresistance; IIK (IκB Kinase), NF-κB, PI3K, AKT, mTOR, JAK, STAT3, Dvl (Dishevlled) APC, GSK-3β, Notch intracellular domain (78).

NF-κB, is a transcription protein that has a pivotal function in several physiological processes such as inflammation, immunological responses, cell proliferation, and longevity (73). In TNBC, NF-κB has been extensively studied because of its involvement in the development and progression of the disease. The activation of NF-κB in TNBC can occur through various mechanisms, and the most common pathway involves the canonical NF-κB pathway. Within this route, several triggers, like inflammatory mediators (e.g., TNF-α), growth factors, or pathogen-associated molecular patterns (PAMPs), initiate the signaling pathway that results in activation of κB kinase (IKK) complex (74, 75). The activated IKK complex phosphorylates the inhibitor of κB (IκB) proteins, which normally bind to NF-κB and keep it sequestered within the cytoplasm. The process of phosphorylating IκB results in its destruction, therefore releasing NF-κB to relocate to the nucleus. Upon entering the nucleus, NF-κB attaches itself to certain DNA sequences called κB sites, located in the promoter regions of target genes and stimulates the transcription of these genes (76). In TNBC, NF-κB activation can occur through various upstream signaling pathways, including those initiated by growth factor receptors (e.g., EGFR), cytokine receptors (e.g., TNF receptor), Toll-like receptors (TLRs), and DNA damage response pathways (77) (**Figure 4**). The transcriptional targets of NF-κB in TNBC include genes involved in cell survival (e.g., Bcl-2, Bcl-xL, cIAP1/2), cell proliferation (e.g., cyclin D1), angiogenesis (e.g., VEGF), invasion and metastasis (e.g., MMP-9, uPA), and inflammation (e.g., IL-6, IL-8, COX-2). By regulating the expression of these genes, NF-κB promotes tumor cell survival, proliferation, angiogenesis, and metastasis, and contributes to the development of an inflammatory microenvironment (79).

#### Monoclonal antibodies against NF-κB pathway

Most treatment strategies concentrate on modifying immune responses or controlling inflammatory pathways that may indirectly influence NF-κB signaling in cancer cells and the tumor microenvironment (78). mAbs such as CD40 agonists, CD137 agonists, and TLR agonists are prospective immunomodulatory approaches that may affect NF-κB signaling via the activation of immune cells and the augmentation of anti-tumor immunity (80).

♣ CD40 Agonistic Monoclonal Antibodies: In TNBC, immune evasion is a considerable problem, and CD40 agonists are under investigation to enhance immunological responses, perhaps rendering tumors more vulnerable to immune cell-mediated eradication (81). CD40 is a costimulatory receptor on immune cells that activates NF-κB activation upon engagement. These antibodies are designed to activate CD40, hence initiating NF-κB signaling and further immune-activating pathways, including cytokine generation and T-cell activation. These reactions may indirectly stimulate NF-κB activation in immune cells (macrophages, dendritic cells) and tumor cells, hence augmenting anti-tumor immunity (82).

♣ CD137 (4-1BB) agonistic antibodies: CD137 is a costimulatory receptor present on T-cells, and its activation has been shown to initiate NF-κB signaling and T-cell activation (83). Agonistic antibodies such as Urelumab are under investigation in clinical studies to elicit immunological responses via NF-κB activation in T-cells. These antibodies indirectly activate NF-κB in cancer cells but may affect the tumor microenvironment, possibly altering NF-κB pathways in immune or cancer cells (84).

♣ TLR (Toll-Like Receptor) Agonists: TLRs are pattern recognition receptors that are crucial in immunological responses and in activation of NF-κB. TLR agonists, like Imiquimod, may stimulate NF-κB in immune cells such as dendritic cells and macrophages, hence enhancing the synthesis of pro-inflammatory cytokines (85). Although Imiquimod is not a monoclonal antibody, it exemplifies the activation of NF-κB via innate immunological signaling, which may be investigated in conjunction with monoclonal antibody treatment (86).

♣ Anti-DR5 (Death Receptor 5) Monoclonal Antibodies: Investigations into TRAIL (TNF-related apoptosis-inducing ligand) receptors and DR5 in TNBC indicates that agonistic antibodies may activate NF-κB in both immune and tumor cells, resulting in apoptosis and increased treatment sensitivity. Nonetheless, a significant portion of this research remains in the preclinical or experimental phase (87, 88).

♣ Targeting NF-κB Pathway Regulators (Inhibition of IκB Kinases and TAK1): In TNBC, dysregulated NF-κB activation fosters inflammation, immunological evasion, and metastasis (89). IKKβ and TAK1 serve as upstream regulators of the NF-κB signaling cascade. Inhibition of these pathways is being investigated to mitigate abnormal NF-κB activation in cancer (90).

### Wnt/β-catenin pathway

The Wnt/β-catenin signaling system is of utmost importance in the genesis, growth, and spread of tumors in TNBC. The Wnt/β-catenin system is a ubiquitous signaling cascade that controls several physiological functions, such as stem cell maintenance, differentiation and cell proliferation. The fundamental constituents of the Wnt/β-catenin cascade are Frizzled receptors, Wnt ligands, and the intracellular protein β-catenin. When Wnt signaling is not present, β-catenin undergoes phosphorylation by a destruction complex composed of casein kinase 1 (CK1), adenomatous polyposis coli (APC), glycogen synthase kinase 3β (GSK-3β) and axin that leads its destruction, hence maintaining low levels of β-catenin in the cytoplasm. The binding of Wnt ligands to Frizzled receptors initiates the signaling pathway that suppresses the expression of the destruction complex. Consequently, β-catenin builds up in the cytoplasm and moves into the nucleus (91). Within the nucleus, β-catenin engages with components of the T-cell factor/lymphoid promoter factor (TCF/LEF) family of transcription factors, resulting in the stimulation of certain genes responsible for cell division and viability (92).

**Figure 5:**
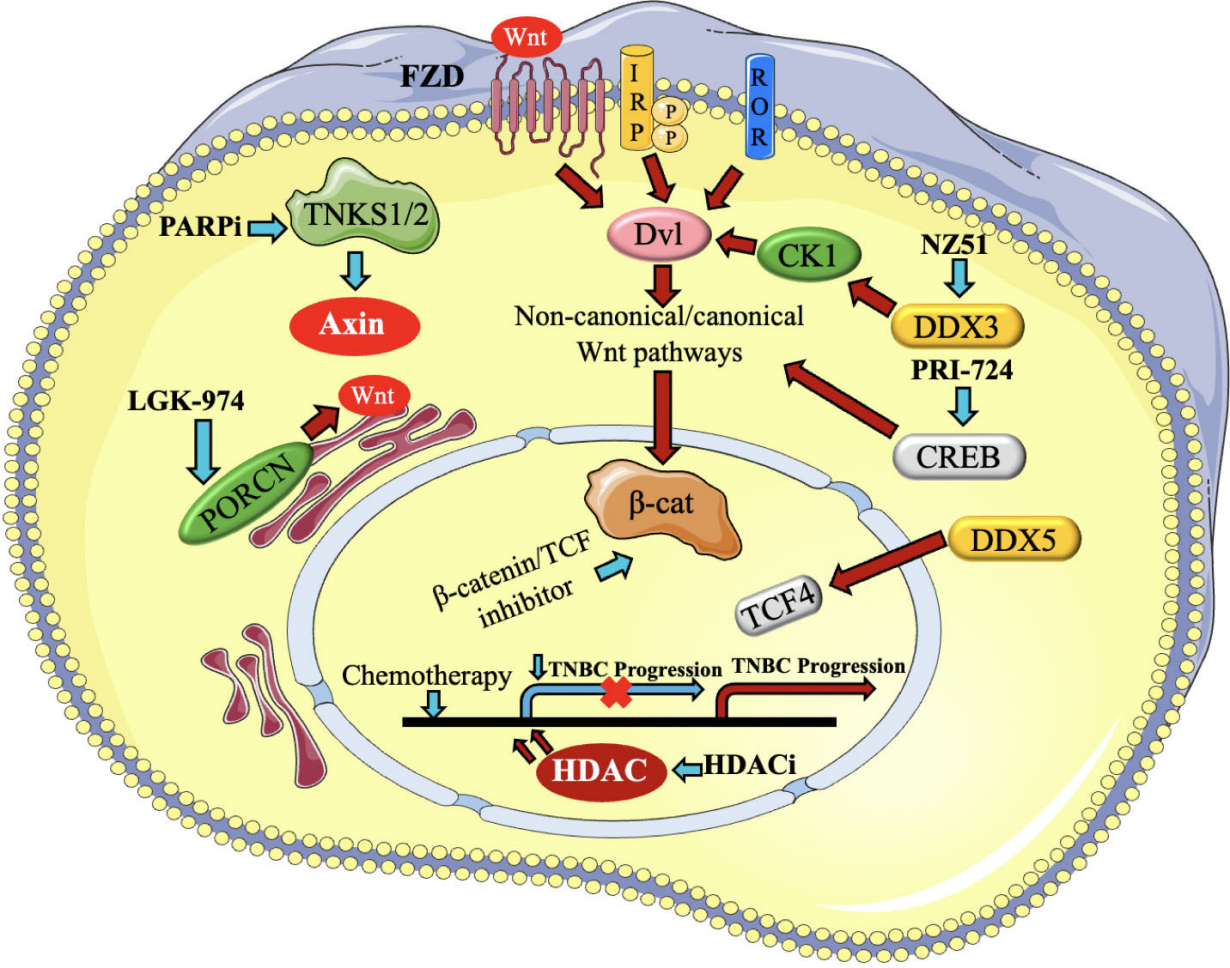
Presents a brief description of the regulators of Wnt signaling that contribute to the advancement of triple- negative breast cancer (TNBC) and the medicines that target them. The activation of canonical and non-canonical Wnt pathways occurs via Fzd, LRP, and ROR receptors. Blue arrows represent the reduction or blocking of Wnt regulators and routes, resulting in the impairment of Wnt target gene transcription, as represented by a yellow cross. On the other hand, red arrows reflect the stimulation of Wnt regulators and pathways (97).

In TNBC, aberrant activation of the Wnt/β-catenin pathway is commonly observed, contributing to tumor growth and progression. Several mechanisms can lead to the dysregulation of this pathway in TNBC, which include Wnt ligand overexpression (93). TNBC cells can produce excess Wnt ligands, which can activate the pathway in an autocrine manner. Changes or modifications in the genes that encode proteins participating in the Wnt/β-catenin cascade, like Axin1, β-catenin (CTNNB1) or APC might disturb the control of β-catenin and result in its stabilization and movement into the nucleus. (94, 95). Various signaling pathways, such as the PI3K/AKT pathway, can activate the Wnt/β-catenin pathway by inhibiting the activity of GSK-3β or promoting the nuclear translocation of β-catenin (96) (**Figure 5**).

The aberrant stimulation of the Wnt/β-catenin cascade in TNBC is associated with increased cell proliferation, decreased apoptosis, enhanced cancer stem cell properties, and resistance to chemotherapy. As a result, focusing on this sequence of events has become a possible treatment approach for TNBC (94). Experimental studies and preclinical models have explored the use of Wnt/β-catenin inhibitors, such as small molecule inhibitors or monoclonal antibodies, to suppress the pathway and inhibit tumor growth. Even though Wnt/β-catenin pathway is an attractive therapeutic target, further research is needed to fully understand its complexity and identify optimal strategies for intervention in TNBC (98, 99).

#### Monoclonal antibodies against Wnt/β-catenin pathway

The Wnt/β-Catenin pathway is often linked to cancer growth, metastasis, and chemoresistance; hence, most research has concentrated on blocking this system rather than activating it directly (97). Ongoing research is investigating monoclonal antibodies (mAbs) that may alter the Wnt/β-Catenin pathway, either indirectly or by targeting upstream components, to influence the tumor microenvironment or immune responses. mAbs specially engineered to activate Wnt/β-Catenin signaling in TNBC are few, with research mostly concentrating on the pathway’s suppression for therapeutic applications (100).

♣ Monoclonal Antibodies that May Influence Wnt/β-Catenin in TNBC

♣ Anti-LRP5/6 (Low-Density Lipoprotein Receptor-Related Protein 5/6) antibodies: LRP5 and LRP6 are co-receptors essential for the activation of Wnt/β-catenin signaling. Inhibiting the interaction between Wnt ligands and LRP5/6 generally suppresses Wnt signaling (101); however, agonistic antibodies directed at these co-receptors may activate Wnt/β-catenin signaling by promoting the formation of the Wnt receptor complex. The advancement of these medicines is mostly in the preclinical stage (102).

♣ GSK3β (Glycogen Synthase Kinase 3 Beta) inhibitors function by inhibiting the kinase GSK3β, which phosphorylates β-catenin, resulting in its destruction and subsequently suppressing Wnt/β-catenin signaling pathway (103). GSK3β inhibitors have been investigated for their potential to enhance β-catenin signaling, which may be used to influence tumor cell characteristics, including stemness and chemoresistance in TNBC (104). Nonetheless, GSK3β inhibitors remain mostly in preclinical or early-phase clinical trials.

### Epidermal growth factor receptor pathway

**Figure 6:**
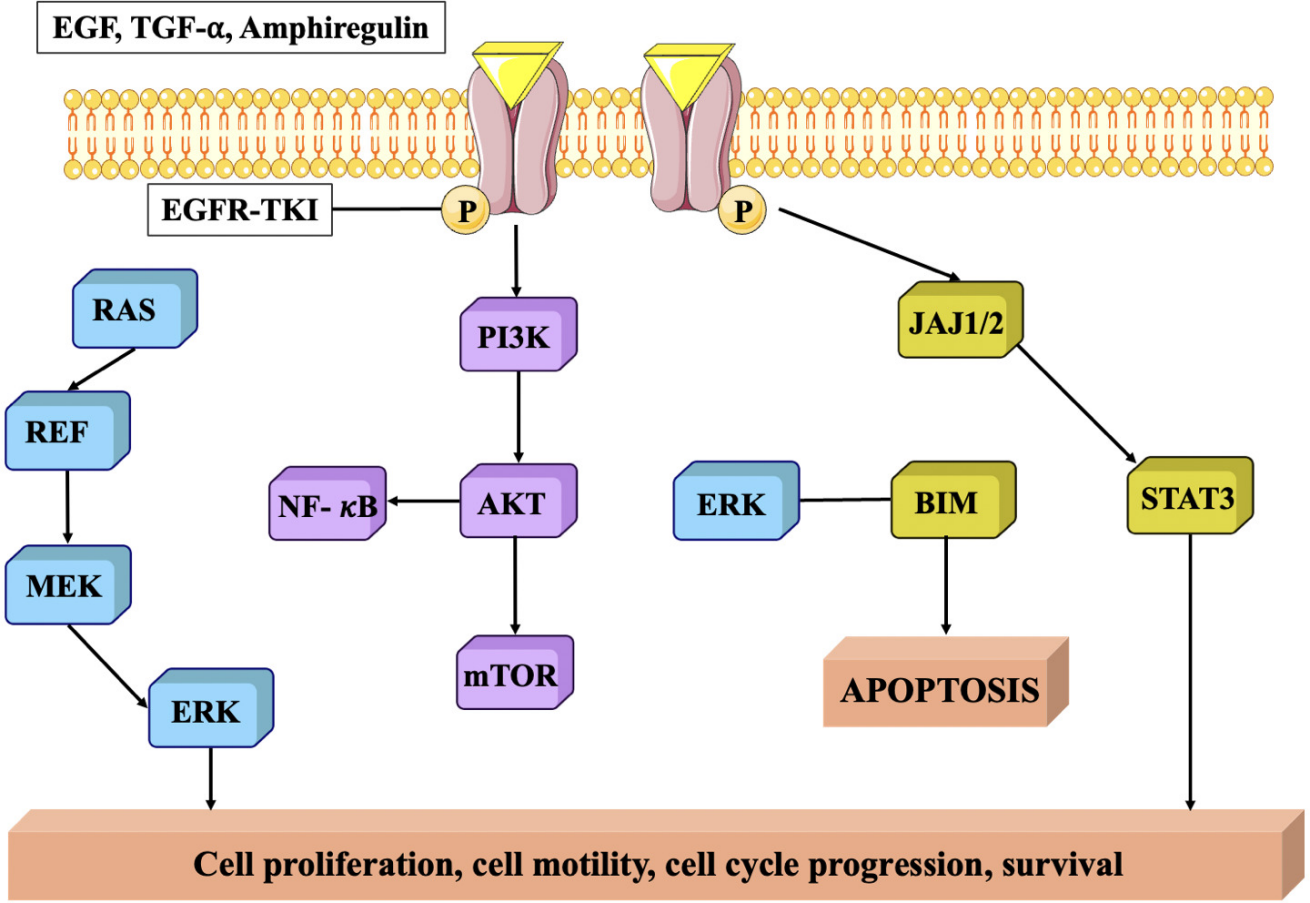
EGFR protein signaling pathway. EGFR is closely related to tumor cell proliferation, angiogenesis, tumor invasion, metastasis and inhibition of apoptosis (108).

EGFR is a member of the ErbB family of receptor tyrosine kinases, and its activation is primarily triggered by the binding of epidermal growth factor (EGF) or other ligands. In TNBC, EGFR is frequently overexpressed which is associated with several mechanisms that contribute to tumor progression and aggressiveness (105). EGFR signaling pathways stimulate cell proliferation and survival, promoting the growth and survival of TNBC cells. EGFR signaling amplifies the aggressive and spreading capabilities of TNBC cells and stimulates the development of matrix metalloproteinases (MMPs), that break down the extracellular matrix and accelerate the advancement and spread of malignant cells (106). EGFR signaling stimulates epithelial-mesenchymal transition (EMT), a mechanism that enables cancerous cells to adopt an enhanced mesenchymal phenotype, hence promoting infiltration and proliferation. It further enhances angiogenesis, by triggering the creation of pro-angiogenic substances including vascular endothelial growth factor (VEGF) (107) (**Figure 6**).

In addition, overexpression of EGFR in TNBC has been linked to resistance to certain treatments. For example, it can activate pro-survival pathways that counteract the effects of chemotherapy drugs and can also interfere with the efficacy of targeted therapies, such as anti-HER2 treatments, by cross-talk between EGFR and HER2 signaling pathways (109, 110).

#### Monoclonal antibodies against EGFR pathway

The majority of monoclonal antibodies (mAbs) directed against EGFR in TNBC have focused on obstructing EGFR signaling to limit tumor proliferation.

♣ Monoclonal antibodies that modulate EGFR signaling in TNBC: EGFR-targeting bispecific antibodies have promise in TNBC, particularly because to the disease’s frequent immune resistance. Bispecific antibodies such as AMG 595 simultaneously target EGFR on tumor cells and CD3 on T cells, facilitating the closeness of immune cells to tumor cells. This medication may indirectly activate EGFR, leads to enhanced immune response via T cell activation having tumoricidal action (111).

♣ Combination therapies using EGFR-targeting monoclonal antibodies, such as Cetuximab or Panitumumab, are being integrated with immune checkpoint inhibitors (e.g., nivolumab, pembrolizumab) to augment the immune response. This strategy indirectly modify EGFR signaling while activating T cells via checkpoint inhibition in TNBC, resulting in enhanced tumor control (108).

♣ Anti-EGFRvIII Antibodies (Mutant EGFR Variant): EGFRvIII is a mutated variant of EGFR that is devoid of the extracellular ligand-binding domain and is often linked to more aggressive malignancies, such as TNBC. mAbs that target EGFRvIII induce receptor activation and subsequent signaling pathways. This constitute a strategy that alters the tumor microenvironment to counteract immune evasion or trigger apoptosis in cells harboring mutant EGFR (112).

### JAK/STAT cascade

**Figure 7:**
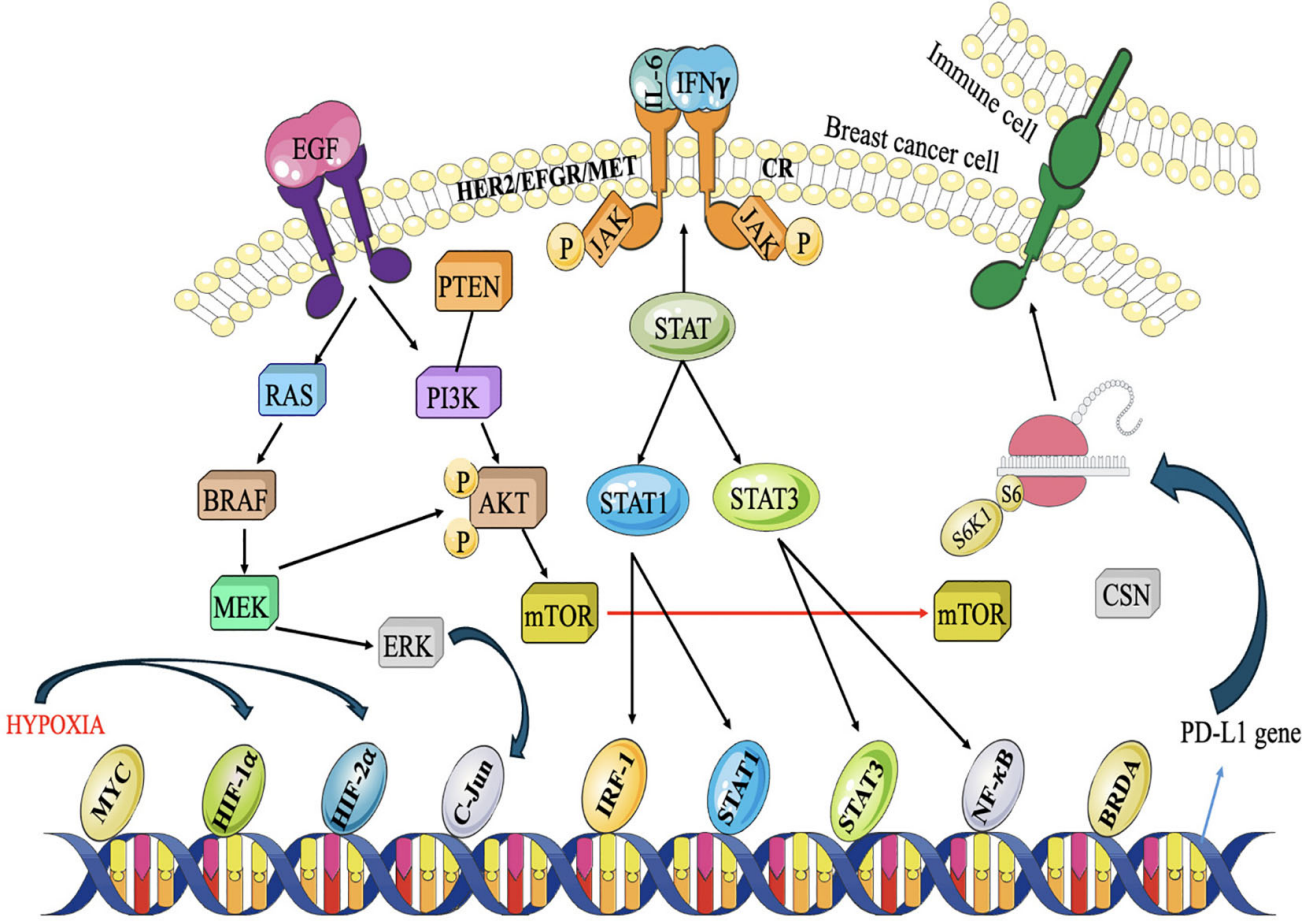
Akt, MAPK, and JAK/STAT pathways collaborate to inhibit the antitumor immune system. Under hy- poxic circumstances caused by Akt signaling, breast cancer cells produce PD-L1 to suppress T-cells. Three major signaling pathways are involved. RTKs stimulate the PI3K/Akt and MAPK pathways, whereas cytokine receptors (CR) are activated by cytokines released into the TME. JAK/STAT signaling is then initiated, recruiting STAT1/3 and other transcription factors to certain gene promoters, including the PD-L1 promoter. Proliferation and metas- tasis are triggered while anti-cancer immunity is deactivated (115).

The JAK/STAT pathway is a signaling cascade that facilitates the transmission of extracellular signals to the cell nucleus, resulting in the expression of certain genes. The composition of this entity includes activator of transcription (STAT) proteins, signal transducer and Janus kinases (JAKs). Cytokines, which are tiny proteins involved in cell signaling, often trigger this signaling pathway (113). In TNBC, mediators like IFN-γ and IL-6 are often increased. The interaction between these mediators and their receptors initiates the expression of JAKs, resulting in the phosphorylation of STAT proteins. These proteins combine to form dimers and are exported to the nucleus, where they attach to certain DNA fragments called responder factors. The engagement of this molecule initiates the procedure of transcribing certain genes responsible for regulating a wide range of biological processes, such as differentiation, survival, development, and migration (114) (**Figure 7**). The JAK/STAT pathway in TNBC may stimulate tumor development by activating the transcription of genes related to cell cycle advancement, inhibition of programmed cell death, and formation of new blood vessels. Additionally, it can also regulate immunological responses (116). Activation of STAT3, stimulates the production of immunosuppressive substances such as programmed death-ligand 1 (PD-L1) and interleukin-10 (IL-10), which hinder the function of defence cells, namely cytotoxic T lymphocytes. This immune evasion mechanism can contribute to tumor immune escape and resistance to immunotherapies (117, 118).

#### Monoclonal antibodies against JAK/STAT signaling pathway

Although direct activation of JAK/STAT signaling with monoclonal antibodies is not a conventional method in the treatment of TNBC, the modulation of this pathway for therapeutic purposes is now a focus of ongoing study (119). Monoclonal antibodies and associated techniques that are currently under development are:

♣ Anti-IL-6 Receptor Antibodies (Indirect Modulation of JAK/STAT): IL-6 is a cytokine that stimulates the JAK/STAT3 system, via STAT3 phosphorylation, which facilitates tumor proliferation and immune evasion in TNBC (120). Tocilizumab, a monoclonal antibody targeting the IL-6 receptor, inhibits the interaction between IL-6 and its receptor, thereby obstructing STAT3 activation. Moreover, inhibiting IL-6 signaling may indirectly activate immunological responses by restoring immune cell function and thereby diminishing tumor immune evasion (115).

♣ Siltuximab an anti-IL-6 mAb that directly neutralizes IL-6, inhibiting its interaction with receptor and consequent JAK/STAT3 activation. Siltuximab generally inhibits IL-6-mediated signaling; which inadvertently stimulate immune system and surmount immunological resistance, resulting in enhanced therapeutic outcomes. This method is still under investigation in conjunction with combo medicines (121).

♣ Anti-GM-CSF monoclonal antibodies: GM-CSF, a cytokine that activates the JAK/STAT pathway and is involved in inflammatory processes and immune cell functionality (122). Gimsilumab is an antibody that targets GM-CSF and may influence JAK/STAT signaling by influencing the inflammatory milieu, including macrophages and dendritic cells, thus impacting the immunological suppression seen in TNBC. This may indirectly bolster the immune response, thereby aiding in the inhibition of tumor development (123).

♣ Anti-CD40 Monoclonal Antibodies (Indirect Modulation of JAK/STAT via Immune Activation). CD40 is a co-stimulatory receptor present on immune cells such as dendritic cells and macrophages. The activation of CD40 may initiate diverse immunological responses and result in the activation of JAK/STAT signaling in immune cells (124). CP-870,893 is an agonistic monoclonal antibody that targets CD40, hence promoting immunological activation and regulating the JAK/STAT pathway in immune cells indirectly (83). This method enhances the immune evasion often seen in TNBC by activating immunological responses and stimulating immune activation via JAK/STAT signaling in immune cells. CP-870,893 is under investigation in conjunction with other therapies to augment antitumor immunity (125, 126).

♣ Directly Targeting JAK1/2 with Monoclonal Antibodies (In Development): JAK1 and JAK2 are essential elements of the JAK/STAT signaling cascade. Oral inhibitors such as Ruxolitinib and Momelotinib are often used in hematologic malignancies, although mAbs that target JAK1/2 remain under development. These antibodies may influence the pathway in certain immunological settings or tumor microenvironments, thereby affecting tumor development or immune evasion seen in TNBC (127).

♣ Monoclonal Antibodies Targeting IL-4 and IL-13: IL-4 and IL-13 are cytokines capable of activating the JAK/STAT6 pathway, particularly within the Th2 immune response. Dupilumab is an antibody that inhibits IL-4 and IL-13 signaling, potentially affecting JAK/STAT signaling in the immune system indirectly. This regulate immune cell function and affect tumor immune evasion (128, 129).

### Notch pathway

The Notch signaling cascade is strongly preserved and is vital in several biological activities, like determining cell destiny, promoting cell growth, facilitating cell specialisation, and ensuring cell survival (130). The Notch pathway is often disrupted in TNBC, resulting in aberrant signaling that plays a role in the pathogenesis and advancement of the illness. The disruption of Notch signaling in TNBC may arise from several causes, including genetic changes, modified expression levels of Notch receptors and ligands, and abnormal stimulation of subsequent signaling elements (131).

**Figure 8:**
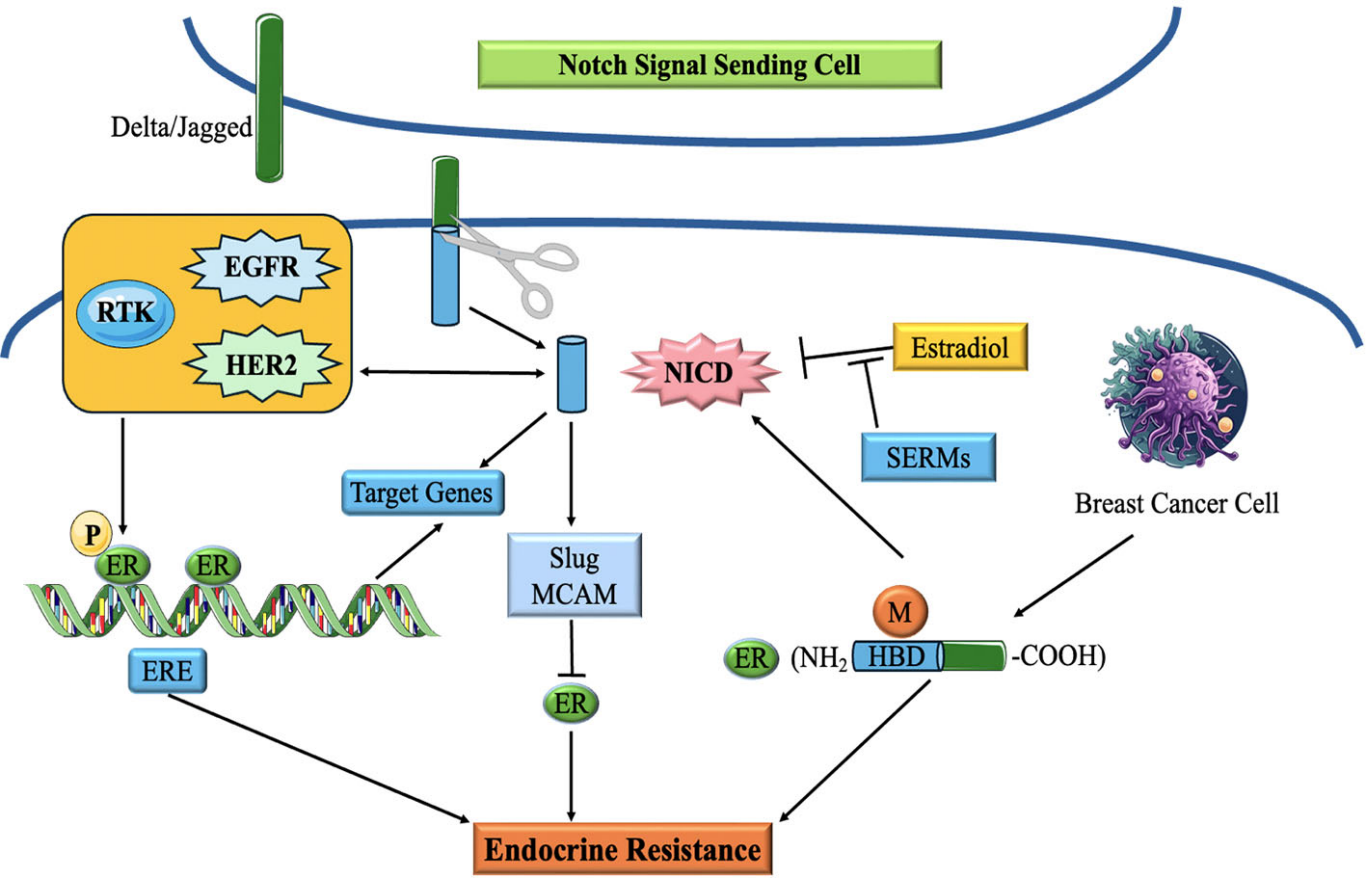
Schematic diagram of the correlation of Notch signaling pathway and ER in breast cancer. Notch Signaling modulates endocrine therapy by cooperating with ER in a complex network (135).

The fundamental constituents of the Notch pathway are Notch receptors (Notch1-4) and their ligands (Jagged1, Jagged2, Delta-like 1, 3, and 4). The Notch pathway is activated by a sequence of proteolytic cleavages. At first, Notch receptors are produced as a solitary polypeptide, subjected to proteolytic cleavage when a ligand binds to it. This process entails the consecutive fragmentation of the receptor by many enzymes, finally liberating the Notch intracellular domain (NICD). After being discharged, NICD moves to the nucleus and combines with other proteins, including co-activators, mastermind-like (MAML) and CSL (CBF1/RBPJκ, Su(H), Lag-1) to create a complex that activates certain genes (132, 133). These targeted genes affect several biological processes and pathways related to TNBC, including cell growth, longevity, EMT, vascular development, and stemness (134) (**Figure 8**).

Notch signaling has been demonstrated to interact with various signaling systems implicated in TNBC, including the EGFR cascade and the Wnt/β-catenin system, hence amplifying its carcinogenic effects. Moreover, abnormal Notch signaling in TNBC has been linked to the initiation of EMT, a mechanism that facilitates tumor penetration and propagation (136, 137).

The activation of Notch signaling enhances the ability of cells to renew themselves and facilitates the development of tumor-initiating cells, also referred to as cancer stem cells (CSCs). The Notch pathway has a role in maintaining CSCs in TNBC by controlling the regulation of genes linked to stem cell characteristics, including Sox2, Nanog and Oct4 (138, 139). The Notch pathway in TNBC may resist conventional chemotherapy agents, such as paclitaxel and doxorubicin and regulate the production of drug efflux pumps, like ABC carriers, which decrease the number of chemotherapeutic drugs that build up within cells. Moreover, its activation can promote the survival of cancer cells by suppressing apoptosis and enhancing DNA repair mechanisms (140).

#### Monoclonal antibodies against Notch signaling pathway

Researches are being conducted on activating the Notch pathway through monoclonal antibodies, with greater emphasis on modulating tumor progression and immune responses rather than directly inducing Notch signaling for therapeutic benefits (135).

♣ Anti-Jagged1 Monoclonal Antibodies: Jagged1 serves as a ligand that activates Notch receptors, particularly Notch1, by interaction with the receptor’s extracellular domain (141). UHD 156, a monoclonal antibody that specifically targets Jagged1, obstructing its interaction with Notch receptors and hence inhibiting its activation leads to diminished tumor proliferation and stem cell properties (142).

♣ Anti-Delta-like Ligands (DLL) Monoclonal Antibodies: Delta-like 4 (DLL4) serves as a ligand that stimulates the Notch signaling pathway, particularly Notch1 and Notch4 (143). PF-06747775, a monoclonal antibody that specifically targets DLL4 and inhibits its capacity to activate Notch receptors, results in diminished Notch signaling. Researchers are also investigating mAbs with other medicines to enhance tumor sensitivity to therapy (144).

♣ Anti-Notch3 mAbs: Notch3 is significantly expressed in TNBC and is essential for the sustenance of cancer stem cells and tumor advancement (145). Investigations are underway about anti-Notch3 mAbs, which may diminish the proliferation and metastatic capacity of TNBC cells, particularly in cases resistant to standard treatment (146). Antibodies targeting Notch3 may also have the ability to influence immune responses inside the tumor microenvironment.

♣ Monoclonal antibodies directed against the Notch intracellular domain (NICD): Following ligand interaction with Notch receptors, the NICD undergoes cleavage and translocates to the nucleus, where it initiates the activation of target genes (147). Research is exploring methods to impede the activation of NICD, linked to tumor stem cell preservation, treatment resistance, and metastasis in TNBC (148).

♣ Notch Agonistic Antibodies (Under Investigation): Although the majority of current research emphasizes the inhibition of the Notch pathway in TNBC (149), there are continuing studies exploring agonistic mAbs that may stimulate Notch signaling in a regulated manner to promote the differentiation of cancer stem cells (150). By activating Notch receptors, these antibodies may compel tumor cells to differentiate, diminishing their stem-like characteristics and enhancing their susceptibility to treatment (151).

### DNA damage response pathway

**Figure 9:**
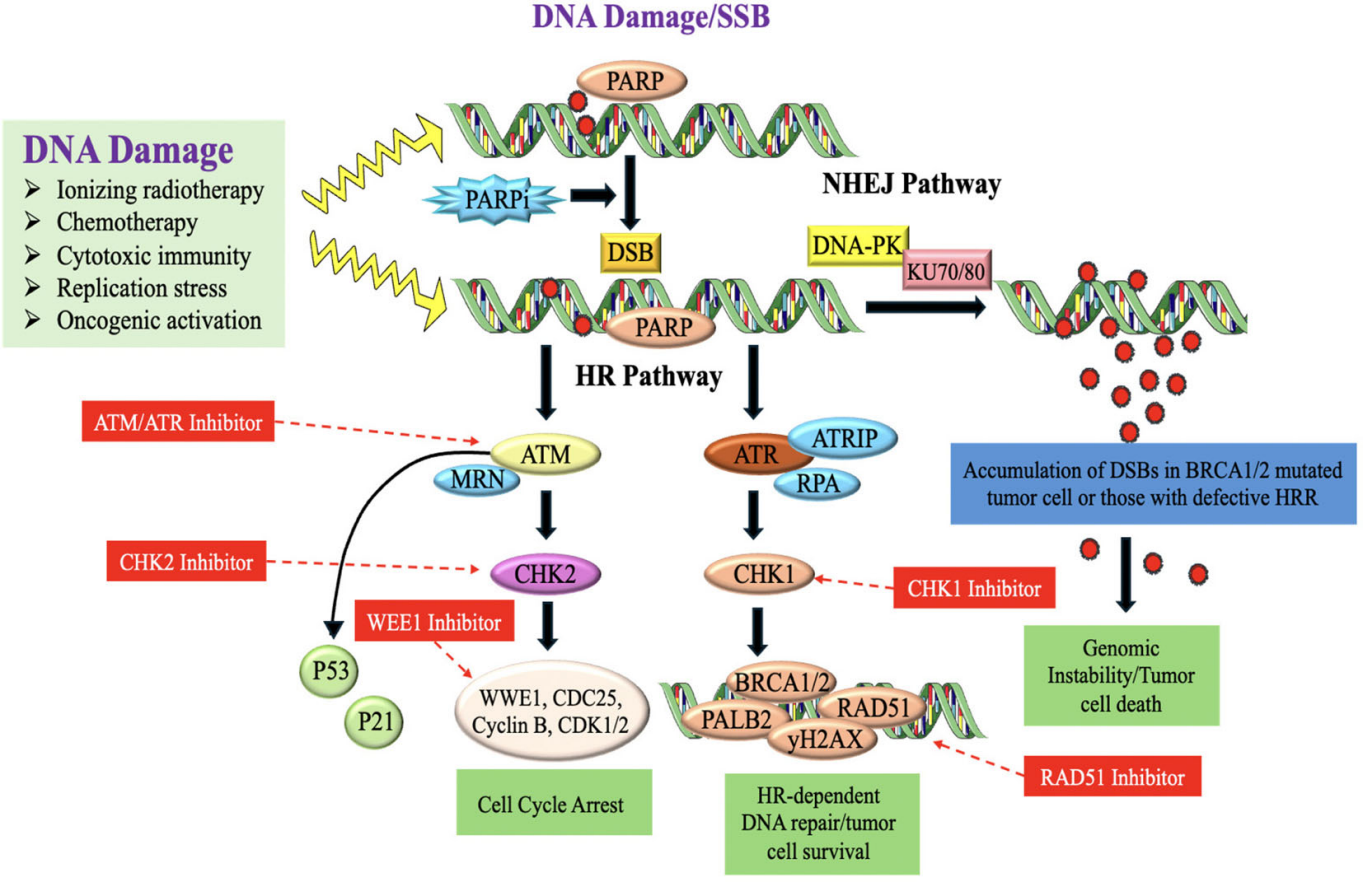
DNA-damaging therapies or endogenous replication dysfunction results in SSBs and DSBs which activate the DDR and repair signaling pathways (157).

DDR is essential for preserving genomic integrity and inhibiting cancer formation. Approximately 15-20% of TNBC cases are associated with germline mutations in the BRCA1 gene. BRCA1 is an important DDR gene involved in DNA repair through homologous recombination (HR) (152). Mutations in BRCA1 impair its function, leading to defective DNA repair and increased genomic instability. These defects make TNBC cells more susceptible to further DNA damage and contribute to tumor development (153). Besides BRCA1 mutations, TNBC can exhibit other defects in HR DNA repair pathways, leading to a state of HRD. HRD causes a reduced capacity to restore DNA double-strand breaks (DSBs) and results in the buildup of genetic changes, which contributes to the development of TNBC tumors (154). TNBC tumors harbouring BRCA1 mutations or (HRD) are more susceptible to medicines that specifically target DNA repair proteins, like poly(ADP-ribose) polymerase (PARP) antagonists (155, 156) (**Figure 9**). DDR dysregulation, including defective DNA repair mechanisms, contributes to the accumulation of these genetic abnormalities which are believed to drive tumor progression and the development of aggressive phenotypes in TNBC (158).

#### Monoclonal antibodies against DNA damage response pathway

Activating the DDR in cancer treatment seeks to provoke genomic instability and death in tumor cells, especially in tumors with deficient DDR pathways (157). Research is continuing to elucidate how mAbs may influence the DDR pathway in the setting of TNBC. This involves targeting essential DDR proteins or augmenting DDR activity in tumor cells to provoke cell death or increase tumor sensitivity to radiation or chemotherapy. Certain medicines seek to stimulate DDR to surmount treatment resistance and induce apoptosis in tumor cells (159).

♣ Monoclonal Antibodies Directed Against DDR Pathways in TNBC:

Anti-ATM Monoclonal Antibodies: ATM is crucial for identifying DNA double-strand breaks and initiating subsequent repair mechanisms. Inhibition of ATM may enhance the susceptibility of cancer cells to DNA-damaging treatments such as radiation or chemotherapy (160). MedI-15 a developing monoclonal antibody that targets ATM, inhibiting its activity and possibly inducing genomic instability in cancers with abnormalities in DDR. Inhibition of ATM in TNBC, particularly in BRCA-deficient tumors, might augment the efficacy of chemotherapy or radiation by impairing repair mechanisms, eventually leading to cell death (161, 162).

♣ Anti-ATR Monoclonal Antibodies: ATR is an essential protein in the DDR system, particularly in the repair of single-strand breaks and the preservation of replication fork stability (163). Ceralasertib, principally a small molecule ATR inhibitor, is under investigation in conjunction with monoclonal antibodies to decrease ATR activity (164). This inhibition may elicit synthetic lethality in BRCA1/2-deficient TNBC by obstructing the repair of replication-associated DNA damage, hence rendering tumor cells more vulnerable to DNA-damaging therapies (165).

♣ Anti-PARP monoclonal antibodies enhance DNA damage response in BRCA-deficient TNBC by targeting PARP, which is important to repair single-strand DNA breaks. PARP inhibitors (PARPi), such as Olaparib and Veliparib, impede the repair of single-strand breaks, resulting in double-strand breaks that need BRCA1/2-mediated repair. In BRCA-deficient TNBC, PARP inhibition induces synthetic lethality, facilitating tumor cell apoptosis (166).

♣ Anti-BRCA Monoclonal Antibodies (Targeting DNA Repair Pathways): BRCA1/2 are essential components of homologous recombination (HR), a vital DNA repair process. mAbs directed against BRCA1/2 seek to alter the tumor’s capacity for DNA damage repair, hence enhancing vulnerability to chemotherapy and radiation. These drugs are being investigation in conjunction with other medicines to elicit synthetic lethality (157, 167).

♣ Anti-Checkpoint Kinase 1 (CHK1) Antibodies: CHK1 is a kinase that modulates the cell cycle checkpoint in reaction to DNA damage. Inhibition of CHK1 may result in cell cycle arrest and heightened DNA damage, hence amplifying the cytotoxic effects of chemotherapy and radiation. Prexasertib a CHK1 inhibitor that may be used in conjunction with mAbs aimed against DDR proteins to enhance the sensitivity of cancer cells (168).

### Hedgehog pathway

The Hedgehog signaling system is essential in the development of TNBC. This signaling system is strongly preserved and controls a range of biological functions in embryonic development and tissue stability. Anomalous stimulation of this mechanism has been linked to the formation and advancement of numerous forms of malignancies, such as TNBC (169, 170). The Hedgehog pathway consists of numerous essential elements, which include Hedgehog ligands (Desert Hedgehog, Indian Hedgehog, and Sonic Hedgehog), transmembrane receptor Patched (PTCH), Smoothened (SMO), and downstream transcription factors belonging to the GLI family (171). When Hedgehog ligands are not present, PTCH prevents SMO from functioning, which leads to the suppression of Hedgehog pathway activity. When Hedgehog ligands connect to PTCH, SMO is no longer inhibited and moves to the main cilium, where it triggers downstream signaling processes (172).

**Figure 10:**
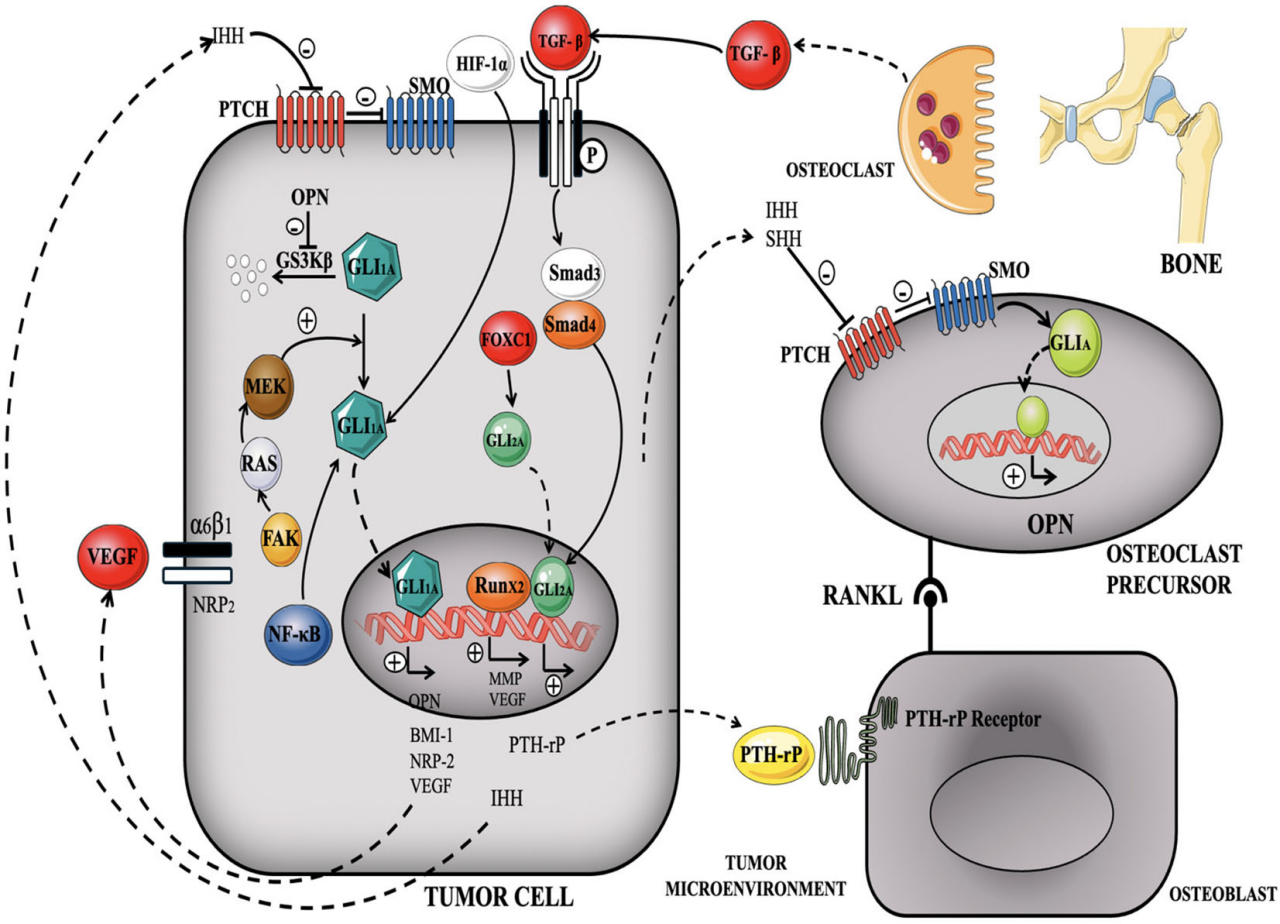
illustrates the triggering of Hedgehog signaling in TNBC, demonstrating the interaction between ligand-dependent and ligand-independent mechanisms (177).

In TNBC, dysfunctioning of the Hedgehog cascade is often linked with increased expression and secretion of Hedgehog ligands, such as Sonic Hedgehog. The activated Hedgehog signaling pathway promotes cancer stemness, cell proliferation, survival, angiogenesis, and metastasis, all of which contribute to TNBC pathogenesis. One of the key mechanisms by which the Hedgehog pathway promotes TNBC is through the activation of cancer stem cells (CSCs) (173, 174). In addition, the Hedgehog system stimulates angiogenesis, by increasing the production of pro-angiogenic proteins such as VEGF (134, 175). Angiogenesis is essential for the progression of tumors since it supplies oxygen and nutrients to support the development of the tumor mass and its spread to other parts of the body. This route also collaborates with other signaling mechanisms, including the Notch pathways and Wnt/β-catenin to facilitate the advancement of TNBC (176) (**Figure 10**).

Amplification of Hedgehog system increases cell cycle regulators, like Cyclin D1 and Cyclin E, resulting in elevated cell division and facilitating tumor development. Furthermore, this network promotes activation of anti-apoptotic proteins, including Bcl-xL and Bcl-2, which hamper apoptosis and enhance the survival of TNBC cells. Research has demonstrated that the amplification of the Hedgehog mechanism in TNBC is linked to the development of resistance to traditional chemotherapy medications by improving the production of drug efflux pumps, like ATP-binding cassette transporters. These pumps help remove chemotherapy medications from cancerous cells resulting in diminished efficacy (64, 178).

#### Monoclonal antibodies against hedgehog pathway

Monoclonal antibodies specially engineered to activate Hedgehog signaling in TNBC are less extensively investigated than inhibitory techniques (177). Research indicates that altering the system to augment or restore Hedgehog signaling may provide therapeutic promise in certain settings, such as overcoming drug resistance or stimulating the differentiation of cancer stem cells (179).

Vismodegib (GDC-0449): Vismodegib is a synthetic, small-molecule inhibitor of SMO, a key component of the hedgehog pathway. It binds and hinders SMO (a trans membrane protein), thus preventing systemic activation of the forward signaling. It results in suppression of Gli-1/2 transcriptional activation which ultimately leads to BCC tumor suppression proving the potential of vismodegib both as a tumoricidal and a tumoristatic candidate (180).

Sonidegib (LDE225): An additional SMO inhibitor now undergoing clinical studies to assess its effectiveness in TNBC. Studies have investigated the use of Sonidegib with chemotherapeutic drugs to address chemoresistance and inhibit tumor proliferation (181).

Anti-Patched1 (PTCH1) Antibodies: PTCH1 is a receptor that typically suppresses SMO in the absence of Hedgehog ligands. Upon binding of Hedgehog ligands to PTCH1, SMO is activated, resulting in downstream signaling. Anti-PTCH1 antibodies may alter the pathway by either obstructing PTCH1 activity to impede tumor growth or by targeting tumor cells that overexpress PTCH1 to possibly reinstate Hedgehog signaling (182).

Anti-GLI Transcription Factor Antibodies: GLI1 and GLI2 are the principal transcription factors that facilitate the ultimate activation of Hedgehog target genes. Anti-GLI antibodies inhibit GLI activation, hence obstructing the production of genes that promote carcinogenesis, chemoresistance, and the maintenance of cancer stem cells. These antibodies are under preliminary development and may be investigated in TNBC to inhibit tumor proliferation and enhance cellular sensitivity to treatment (183).

Anti-Hedgehog Ligand (Shh) Antibodies: Shh is a principal ligand that activates the Hedgehog pathway via its interaction with PTCH1. Anti-Shh mABs may inhibit this ligand’s capacity to activate the pathway, hence obstructing downstream signaling events that contribute to tumor growth and chemoresistance (184).

### MET pathway

**Figure 11:**
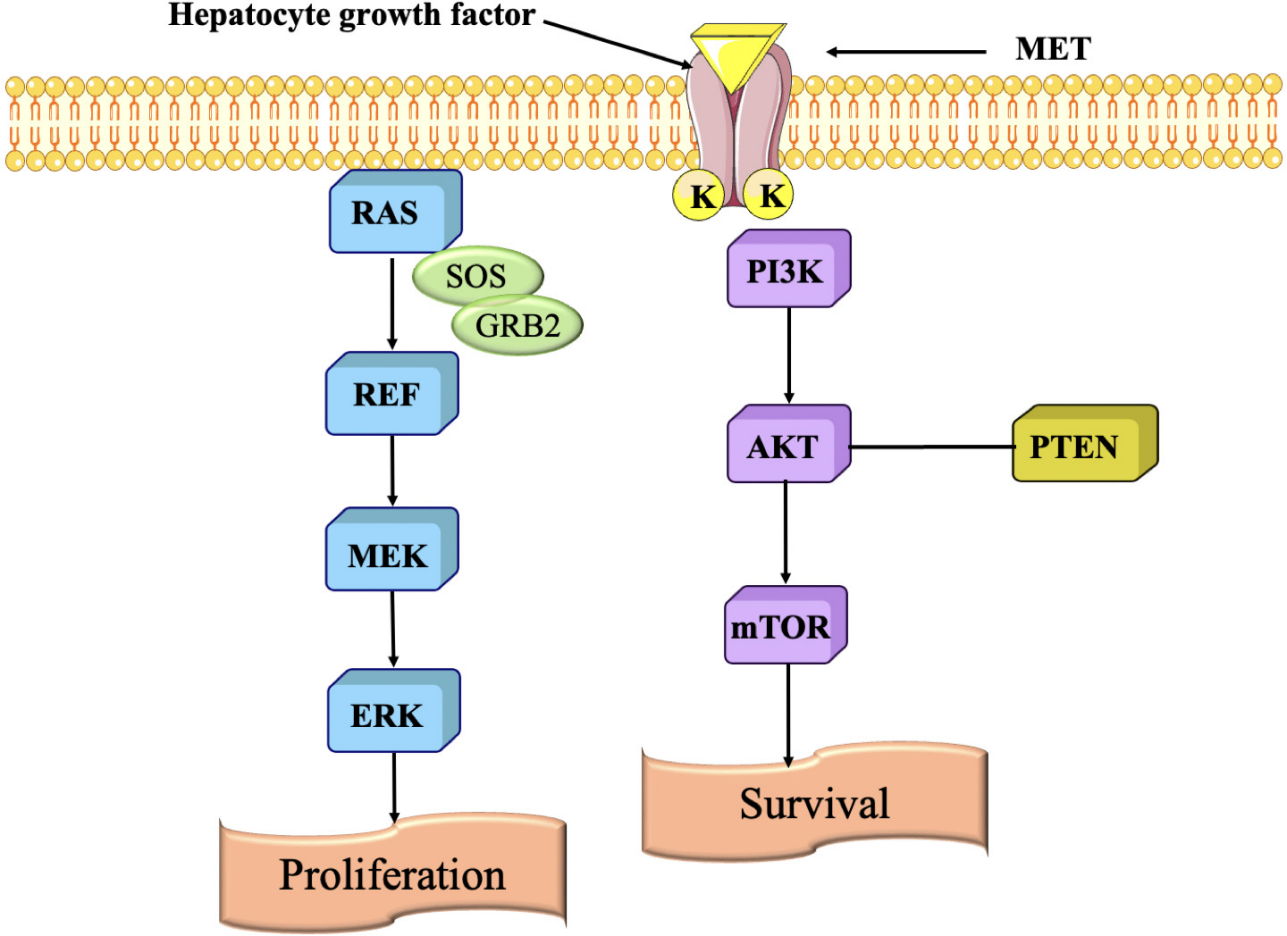
Signaling pathway are regulated by scatter factor (HGF) binding to MET resulting in activation of various pathways (186).

The MET pathway is primarily involved in cell growth, survival, motility, and invasion. It is activated by the hepatocyte growth factor (HGF), which binds to the MET receptor tyrosine kinase. When HGF binds to MET, it triggers a signaling cascade that leads to various cellular responses (185). The MET pathway in TNBC exhibits irregularity, which is associated with cancer growth and metastasis (**Figure 11**). The MET pathway enhances cellular proliferation and survival in TNBC (187). The signaling pathway triggers cell cycle progression, allowing cancer cells to grow and divide uncontrollably. It amplifies the capacity of TNBC cells to invade and spread, promoting cell migration, infiltration into neighbouring tissues, and the formation of metastatic tumors in organs like the lungs, liver, and bones (188). This signaling pathway controls the regulation of genes that are engaged in the course of EMT, which is linked with an upsurge in invasiveness and metastasis. The MET pathway is also crucial in facilitating new blood vessel formation. TNBC tumors rely on angiogenesis to receive adequate blood supply for their growth and survival. The MET pathway influences the production of pro-angiogenic factors, facilitating the formation of blood vessels within the tumor microenvironment (189).

#### Monoclonal antibodies against MET pathway

Monoclonal antibodies that activate the MET pathway remain largely unexplored. The majority of research concentrates on obstructing MET signaling because of its involvement in facilitating tumor advancement and medication resistance (190).

♣ Monoclonal Antibodies Directed Against MET in TNBC

Inhibitory Anti-MET Monoclonal Antibodies: Although MET activation is often linked to carcinogenesis, mAbs that block MET (such as onartuzumab, savolitinib, and capmatinib) are now undergoing clinical trials for TNBC (186).

♣ Onartuzumab (MetMAb) a monoclonal antibody that targets the MET receptor, inhibiting the binding of its ligand HGF and obstructing MET activation. This antibody has been examined in early-phase clinical studies for MET-positive TNBC to evaluate its efficacy in inhibiting tumor development and chemoresistance. Onartuzumab is often used in conjunction with other medicines, including chemotherapy (191).

♣ Savolitinib (AZD6094) a MET inhibitor that obstructs the MET receptor. Clinical trials are under progress to assess its efficacy in inhibiting tumor proliferation and overcoming resistance in MET-driven TNBC (192).

♣ Anti-HGF Monoclonal Antibodies: Hepatocyte growth factor (HGF) serves as the ligand that activates MET, and anti-HGF monoclonal antibodies are under investigation to inhibit this activation (192). By inhibiting the binding of HGF to MET, these antibodies may impede tumor proliferation and invasion in MET-dependent malignancies such as TNBC (193).

### HER2 signaling (Amplification)

**Figure 12:**
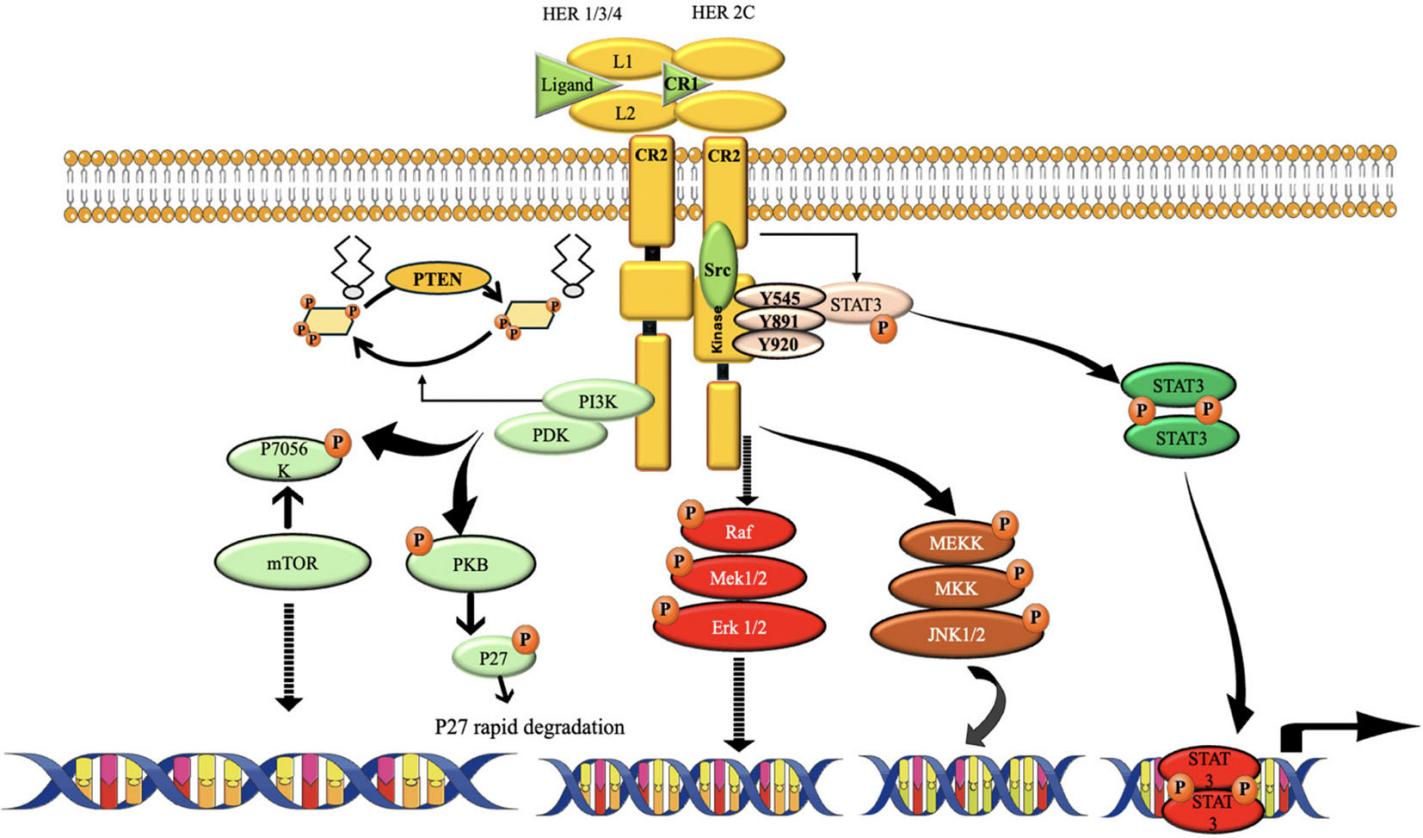
The route of HER2 signaling. The activation of HER2 requires the receptors to form homodimers or heterodimers. It promotes cell growth, proliferation, and survival, among other actions, by activating a wide variety of downstream cascades. One of the most extensively researched pathways triggered by HER2 is PI3K/Akt. The major positive regulator of cellular metabolism, mTOR, is activated when PI3k/Akt is. And via activating the Ras/Raf and MEK pathways, HER2 may promote the migration and development of cancer cells (196).

“Although TNBC lacks HER2 amplification by definition, compensatory signaling through EGFR and other HER family members can activate downstream PI3K/AKT and MAPK pathways, functionally mimicking HER2-driven oncogenesis.” (194, 195). These systems control critical cellular functions, including cell cycle progression and cell survival (**Figure 12**). This pathway regulates key cellular processes involved in cancer progression, including cell cycle regulation and protein synthesis. The overexpression of HER2 can also lead to the dysregulation of the MAPK pathway. Stimulation of this route may enhance the growth, infiltration, and formation of new blood vessels in cells, hence promoting the possessive nature of the TNBC (106, 197).

#### Monoclonal antibodies against HER2 pathway

While HER2-targeted medicines such as trastuzumab (Herceptin) are primarily used for HER2-positive breast cancer, novel mAbs and targeted therapies are being investigated for HER2-low or HER2-mutant TNBC to activate or modify HER2 signaling for therapeutic advantage. Certain techniques focus on targeting the HER2 receptor to elicit downstream signaling for specific results, such as differentiation or death in certain settings (198).

♣ Margetuximab (MGAH22) a recombinant monoclonal antibody that specifically targets HER2 and has enhanced immune-effector capabilities. It is intended to induce antibody-dependent cellular cytotoxicity (ADCC), resulting in the annihilation of malignant cells (196). Margetuximab is being evaluated for its efficacy in enhancing immune responses in HER2-positive tumors and may enhance HER2 signaling in HER2-low TNBC, facilitating immunogenic cell death and improving tumor detection by the immune system (196, 199).

♣ Pertuzumab (Perjeta) a monoclonal antibody that inhibits the dimerization domain of HER2, obstructing its interaction with other HER family receptors. This inhibits HER2 signaling and triggers apoptosis (200). It is often used in conjunction with Trastuzumab for HER2-positive malignancies. In HER2-low TNBC, Pertuzumab is being investigated to enhance immune response and induce apoptosis when used in conjunction with Trastuzumab and chemotherapy (198).

♣ HER2-Targeted Biologic Therapies with Modulatory Effects: Trastuzumab-Emtansine (T-DM1) is an antibody-drug conjugate (ADC) that integrates Trastuzumab with the cytotoxic compound emtansine. This medication is intended to provide chemotherapy specifically to tumor cells that express HER2. T-DM1, primarily used in HER2-positive breast cancer, is being investigated for its efficacy in HER2-low TNBC. It may activate HER2 signaling and enhance tumor cell apoptosis by selectively targeting cells with low HER2 expression (201).

♣ Sacituzumab Govitecan is an antibody-drug conjugate (ADC) that specifically targets Trop-2, a cell surface protein expressed in TNBC (202). Research is now being conducted to examine how HER2-targeting antibody-drug conjugates, such as Sacituzumab, may activate signaling pathways that potentially amplify their efficacy in HER2-low cancers (203). It is being evaluated in conjunction with HER2-targeted therapy to explore the potential for synergistic activation of signaling pathways and tumor suppression by concurrent targeting of HER2 and Trop-2 (204).

♣ Bispecific Antibodies: Blinatumomab (Blincyto) is a bispecific T-cell engager (BiTE) that attaches to CD3 on T cells and HER2 on tumor cells, therefore eliciting T-cell-mediated cytotoxicity (205). In HER2-low TNBC, Blinatumomab may augment the immune system’s capacity to identify and eliminate HER2-expressing tumor cells while activating HER2 signaling to promote tumor regression (206).

♣ Current and Upcoming Clinical Trials in HER2-Targeted Therapy for TNBC:

The IMpassion130 trial examines the efficacy of Atezolizumab (anti-PD-L1) in conjunction with nab-paclitaxel (chemotherapy) in HER2-negative TNBC, including HER2-low tumors in the subgroup analysis. The function of HER2 signaling in relation to immunotherapy is now under assessment in these individuals (207).

♣ The HER2CLIMB study: This clinical trial assesses Trastuzumab-Emtansine (T-DM1) in patients with HER2-low TNBC, investigating the efficacy of this HER2-targeted antibody-drug conjugate in tumors with reduced HER2 expression (208).

**TGF-β (Transforming Growth Factor-beta) Pathway:**

**Figure 13:**
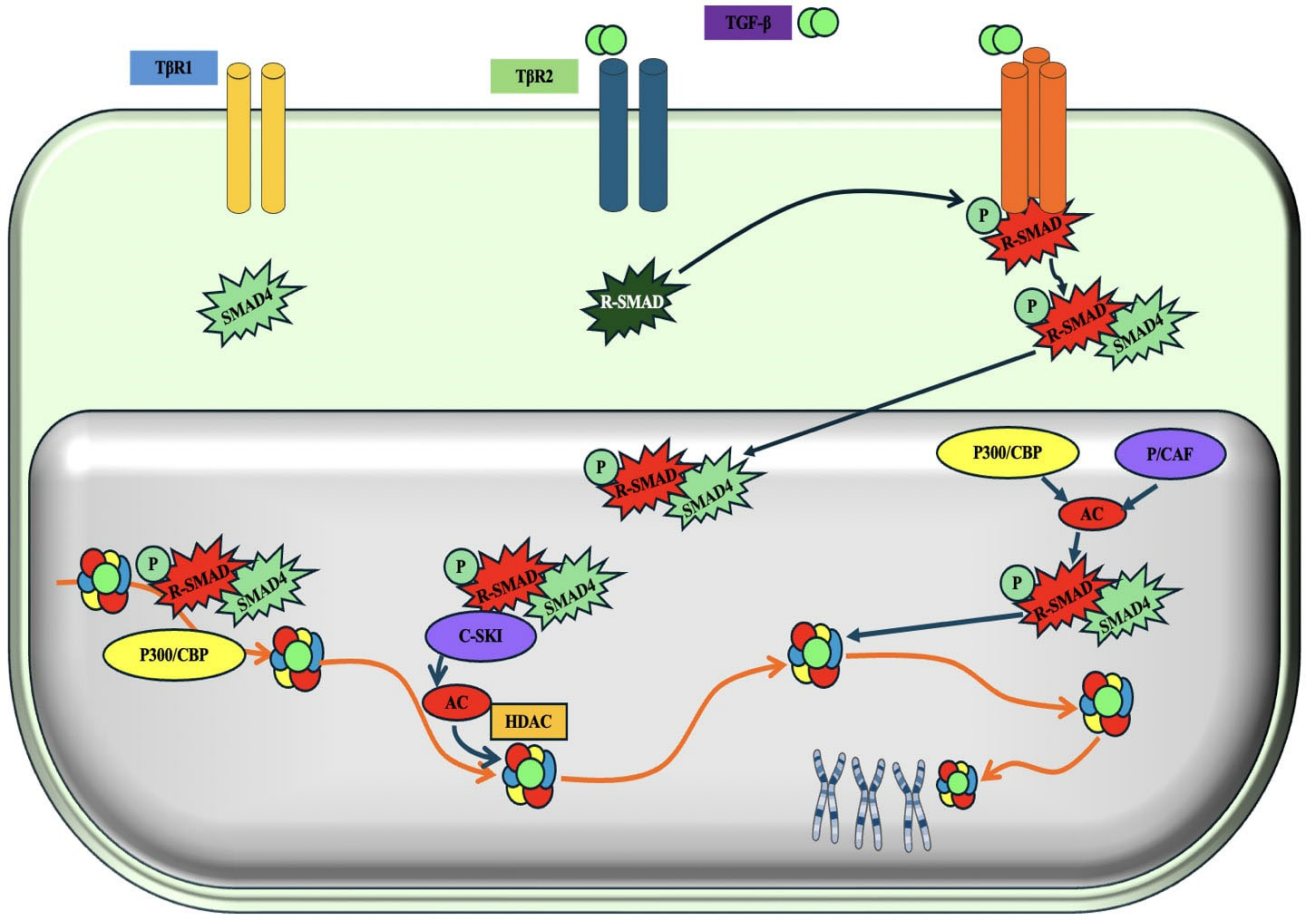
Schematic representation of the TGF-β (Transforming growth factor-β/ SMAD (SMA and MAD related protein)-induced transcriptional response mediated by coactivators and corepressors. 'P' in yellow circles indicates phosphorylation. Arrows denote the addition of a modification or transfer of a protein complex and the dotted arrow represents the reverse of this. 'Ac' indicates acetylation (213).

The TGF-β pathway is very influential in TNBC. This pathway is intricate and plays a role in several biological processes, such as cell proliferation, differentiation, death, and immunological control. The composition of this entity includes TGF-β receptors, TGF-β ligands, and downstream signaling effectors (209, 210). TGF-β ligands, namely TGF-β1, TGF-β2, and TGF-β3, are proteins that are released and attached to TGF-β receptors, which consist of type I and type II (TGF-βR) receptors. TGF-βRII phosphorylates and promotes TGF-βRI, which initiates the signaling cascade downstream (211). Once activated, TGF-βRI initiates a signaling cascade through the Smad proteins. Smad2 and Smad3 are phosphorylated by TGF-βRI and form a complex with Smad4 (212) (**Figure 13**). This intricate molecule moves into the nucleus, where it controls the process of transcribing certain genes. TGF-β functions as a tumor inhibitor in the first phases of breast cancer by impeding cell growth, triggering cell cycle arrest, and facilitating programmed cell death. Additionally, it contributes to the maintenance of tissue homeostasis. Nevertheless, at the later stages of TNBC, the TGF-β cascade may experience dysregulation, resulting in the elimination of its ability to control the tumor growth (214, 215).

#### Monoclonal antibodies against TGF-β pathway

Clinical studies are investigating mAbs that target the TGF-β pathway to either inhibit or regulate it, aiming to boost tumor suppression, immune activation, or improve the effectiveness of other treatments (216).

♣ Investigation of mAbs Targeting the TGF-β Pathway in TNBC

♣ Monoclonal Antibodies Against TGF-β: Fresolimumab (GC1008) a mAb targeting TGF-β that binds to all three isoforms (TGF-β1, TGF-β2, and TGF-β3), inhibiting their activation and signaling pathways (217). Fresolimumab seeks to restore normal immune function and diminish the tumor-promoting effects of TGF-β in cancer by inhibiting TGF-β signaling. In TNBC, Fresolimumab is undergoing evaluation with chemotherapy and immune checkpoint inhibitors to augment the immune system’s capacity to identify and eliminate tumor cells (213).

♣ M7824 is a bifunctional fusion protein that concurrently binds TGF-βRII and PD-L1. This combinatorial strategy obstructs TGF-β signaling and suppresses immunological checkpoint signaling (PD-L1), resulting in enhanced anti-tumor immunity (218). M7824 is being assessed in TNBC to enhance the immune response against the tumor while inhibiting the immune-evasive processes mediated by TGF-β (219).

♣ Trabedersen (AP 12009) is a TGF-β2-specific antisense oligonucleotide that targets the TGF-β2 gene at the RNA level, inhibiting the synthesis of TGF-β2 and mitigating its impact on tumor growth and immune suppression (220). It has been investigated for its capacity to inhibit TGF-β2 expression in TNBC, especially in instances when TGF-β2 significantly contributes to tumor spread and immune evasion (221).

### Focal adhesion kinase pathway

**Figure 14:**
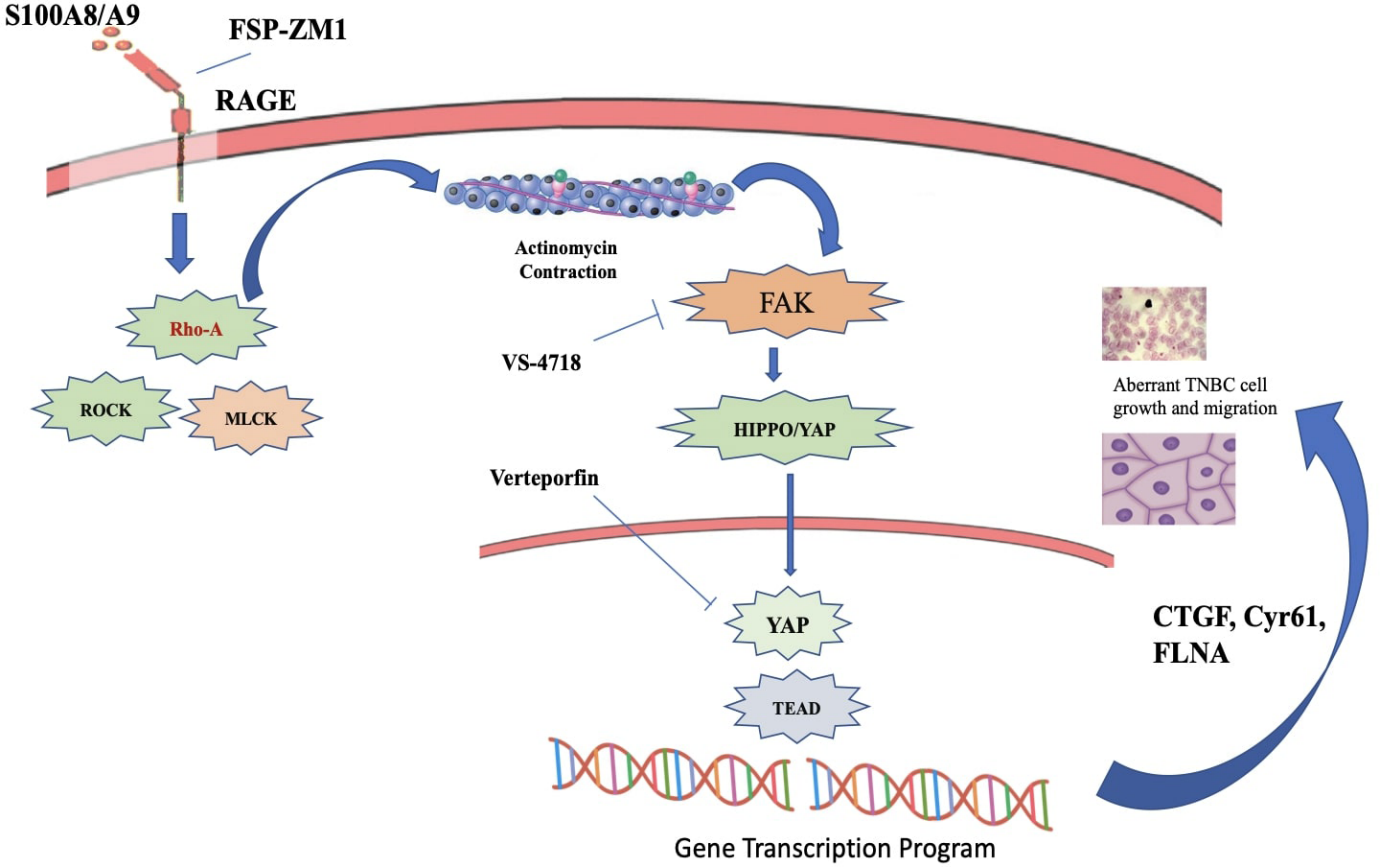
S100A8/A9-RAGE-FAK-YAP signaling in TNBC cells. A cartoon depicting the proposed S100A8/A9-RAGE-FAK-YAP transduction network in TNBC cells Targeting RAGE along with FAK/YAP-dependent transcriptional programs may disable the growth and migration of TNBC cells (226).

FAK, a non-receptor tyrosine kinase, is essential for integrin-mediated signaling and the processes of cell adherence, growth migration, and longevity. FAK participates in signaling pathways that govern these activities, and its abnormal regulation has been linked to the development of several malignancies, such as TNBC (222, 223). FAK governs the generation and degradation of focal adhesions, which are dynamic structures that facilitate cell attachment to the extracellular matrix (ECM). FAK enhances cell migration by initiating downstream signaling pathways, such as the PI3K/Akt and MAPK pathways, resulting in heightened cell motility and invasion. (224). In TNBC, aberrant FAK signaling can enhance the migratory and invasive properties of cancer cells, facilitating metastasis. Its activation promotes cell survival and proliferation through various mechanisms. It can initiate pro-survival signaling pathways, such as the PI3K/Akt and STAT3, which play a role in promoting cell survival and preventing apoptosis (225) (**Figure 14**). FAK may also stimulate cancer development by facilitating angiogenesis, that provide nutrition and oxygen to the developing tumor. It also has been implicated in EMT regulation by activating transcription factors such as Snail, Slug, and Twist, which promote EMT-associated changes in cell morphology and behaviour (222). FAK signaling has been linked to resistance to many anti-cancer treatments, like targeted therapies and chemotherapy by promoting cell survival and reducing the efficacy of cytotoxic drugs. Additionally, FAK can modulate signaling pathways that mediate resistance to targeted therapies, such as HER2 inhibitors or PARP inhibitors (227).

### Pharmacotherapeutic therapies against FAK signaling pathway

Research on various pharmacotherapeutics therapies especially aimed at FAK signaling in TNBC is ongoing, with several strategies focusing on either directly targeting FAK or modifying pathways associated with FAK activation (228). Several therapies and methodologies under investigation that may influence FAK signaling in TNBC.

♣ Defactinib (VS-6063) a small molecule inhibitor that specifically inhibits the kinase activity of FAK and has been extensively researched in conjunction with other therapies, including immune checkpoint inhibitors and chemotherapy. It particularly inhibits FAK signaling and undermines the tumorigenic effects of FAK activation in cancer cells (229).

### Signal transducer and activator of transcription 3 pathway

It is a transcription component that has a crucial function in the development of TNBC. The STAT3 pathway is activated by various cytokines, growth factors, and oncogenic signaling pathways (230). Upon activation, STAT3 relocates to the nucleus and controls the transcription of target genes associated with cell survival, proliferation, angiogenesis, immunological evasion, and metastasis (226).

**Figure 15:**
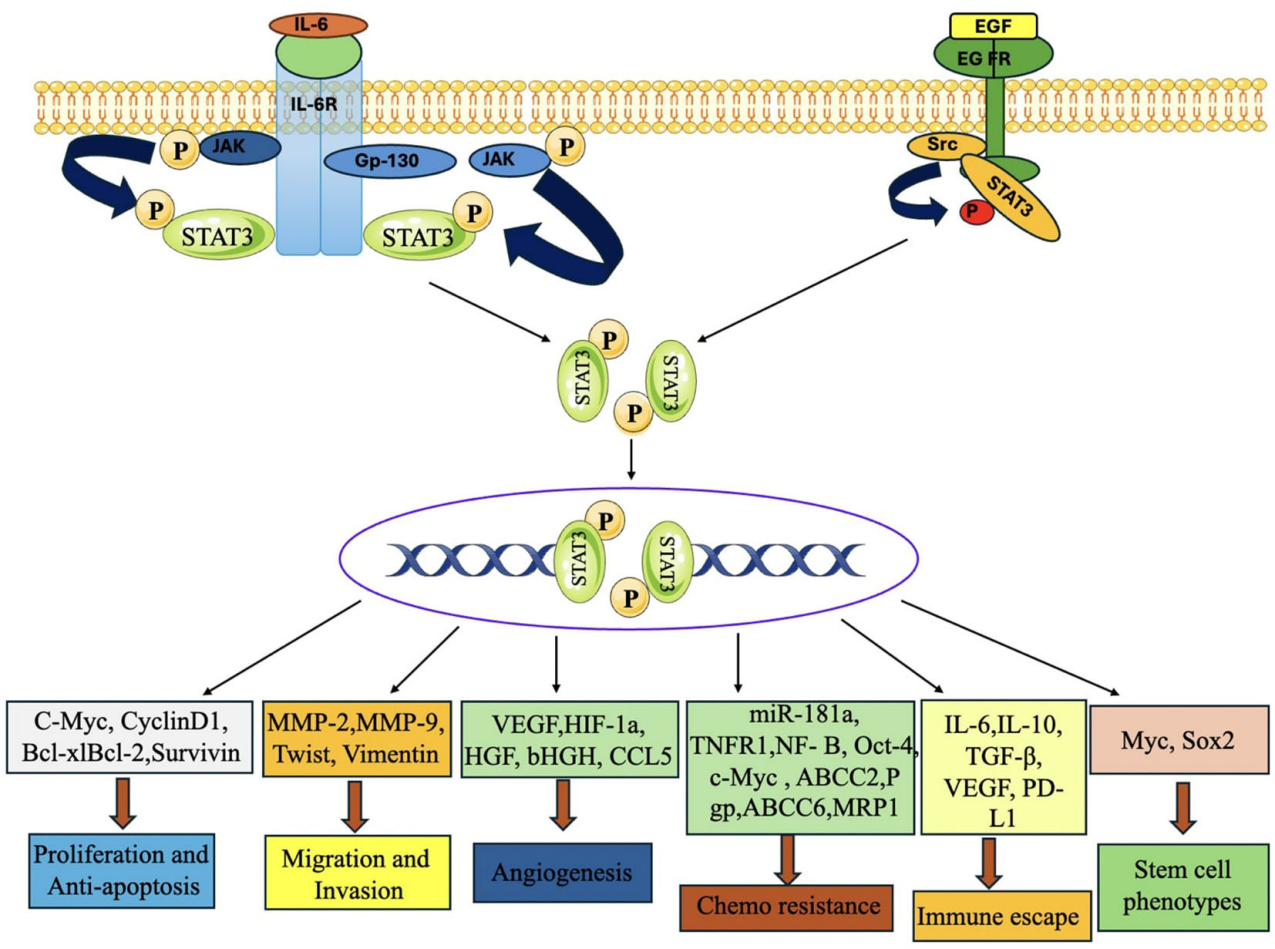
STAT3 signaling activation enhances tumor development, metastasis, chemoresistance, immunological evasion, and stemness in TNBC. When the upstream regulators are activated, STAT3 is phosphorylated, dimerized, and transported into the nucleus, where it activates the transcription of target genes that control cell proliferation, anti-apoptosis, migration, invasion, angiogenesis, chemoresistance, immune escape, stem cell phenotypes, and autophagy (231).

The STAT3 pathway is often activated in TNBC, leading to abnormal gene expression that promotes cell cycle progression and suppresses apoptosis. It upregulates cyclin D1, cyclin-dependent kinase 4 (CDK4), and survivin, which promote cell proliferation and suppress cell death (116). STAT3 stimulates the production of pro-angiogenic substances, including fibroblast growth factor (FGF), interleukin-8 (IL-8) and VEGF (**Figure 15**). Additionally, it stimulates EMT and also hinders the body’s defences by impeding the activity of immune cells, including T cells and natural killer (NK) cells. It enhances the production of immunological checkpoint components, such as programmed death-ligand 1 (PD-L1), which helps in avoiding detection by the immune system (232).

Activation of STAT3 has been linked to opposition to chemotherapy treatments via the promotion of drug efflux pumps, like P-glycoprotein (P-gp). These pumps decrease the buildup of pharmaceuticals within cells, hence contributing to resistance to therapy (233).

#### Combination strategies (monoclonal and non- monoclonal antibodies) against STAT3 pathway

Monoclonal antibodies that target the STAT3 pathway constitute a promising study domain for TNBC, especially since STAT3 plays a critical role in immune evasion and tumor advancement. Despite some medicines being in preliminary research phases, combination therapies using monoclonal antibodies and STAT3 inhibitors show significant promise in both preclinical and clinical studies (234).

♣ Monoclonal Antibodies Directed Against the STAT3 Pathway in TNBC.

The combination of N-803 (an IL-15 superagonist) with the anti-STAT3 mAb (CXXC5) is under investigation for its ability to target and inhibit the STAT3 pathway. N-803 promotes immunological activation, while CXXC5 inhibits the phosphorylation of STAT3. These drugs seek to inhibit STAT3 phosphorylation to reinstate immune surveillance, diminish cancer cell proliferation, and enhance tumor sensitivity to alternative treatments, such as immunotherapy (235).

♣ CDDO-Me, a synthetic STAT3 inhibitor, is now being evaluation for its ability to augment the effectiveness of mAb therapy, especially in metastatic TNBC (236). The objective is to diminish STAT3 activity, which often results in immune evasion and tumor persistence. This method remains in the preclinical phase, although it has potential as a combinatorial treatment with immune checkpoint inhibitors or other medicines targeting STAT3 suppression in TNBC (237).

♣ S3I-201 (STAT3 Inhibitor) Combined with Chemotherapy or Immunotherapy: S3I-201 a small molecule inhibitor of STAT3 has shown preclinical efficacy in many malignancies, including TNBC. S3I-201, a non-monoclonal antibody, directly inhibits STAT3 signaling by obstructing its phosphorylation and nuclear translocation (231). Researchers are evaluating this inhibitor with chemotherapy and immunotherapy (e.g., anti-PD-1/PD-L1 inhibitors) aiming to evaluate the safety, tolerability, and effectiveness of S3I-201, as well as its capacity to enhance the sensitivity of TNBC cells to other treatments, including mAbs (235).

### Hippo pathway

**Figure 16:**
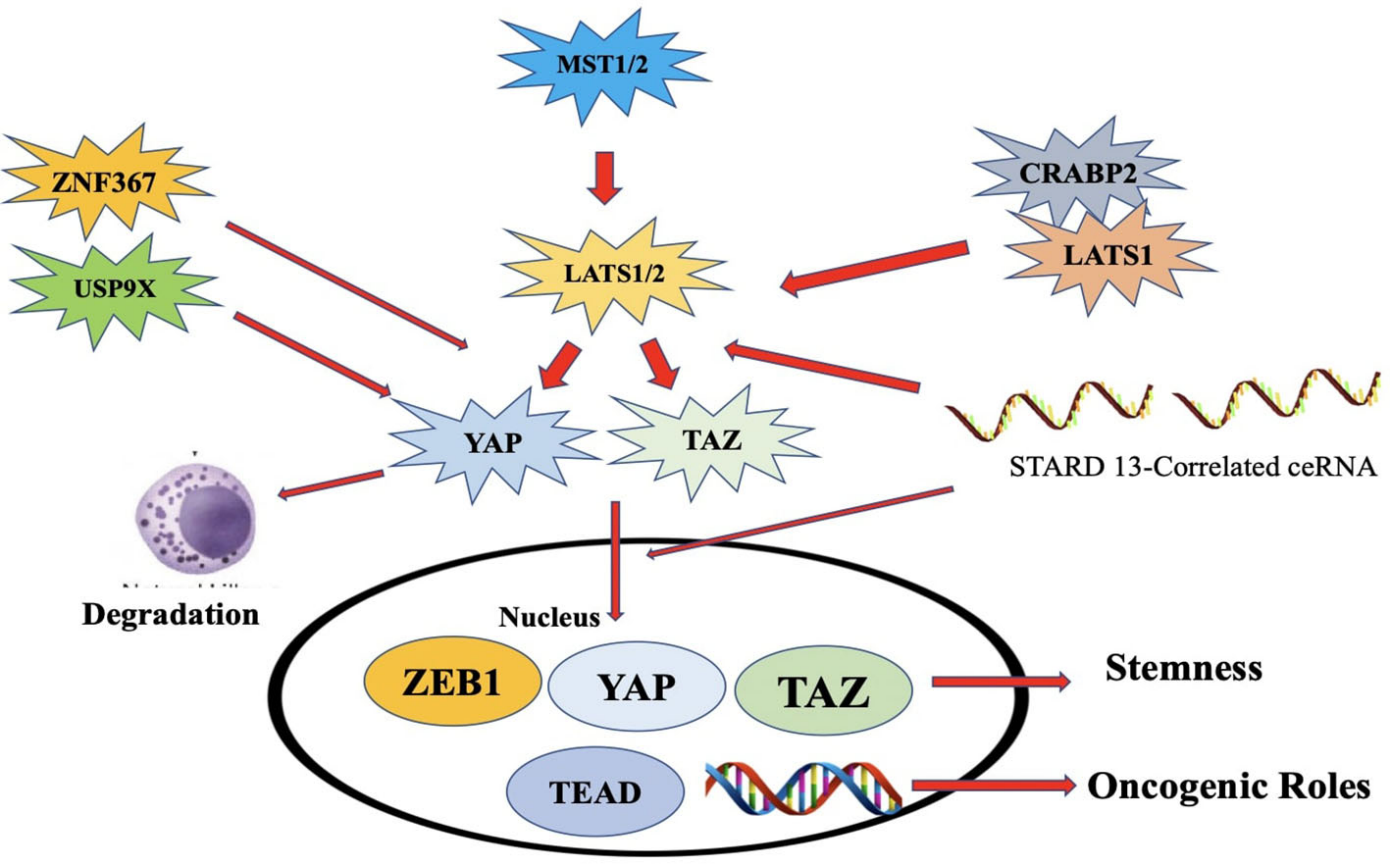
An illustration of the Hippo pathway's significance in ER+ breast cancer. Overexpression of ZNF367 promotes metastasis and stimulates Hippo/YAP signaling via blocking LATS. Overexpression of USP9x promotes cell proliferation by deubiquitinating and stabilizing YAP1.NE and EPI suppress breast cancer by rapidly phos-phorylating YAP and retaining it in the cytoplasm. ZEB1, a transcriptional activator, interacts with YAPI to in-crease transcription. The connection between LATS1 and CRABP2 decreases LATS1 ubiquitination, which sup-presses cell invasion. The STARD13-correlated ceRNA network controls TAZ distribution and may decrease the stem cell nature of breast cancer by upregulating LATS1/2 [248].

The Hippo pathway is a well-preserved signaling route that regulates the size of organs and the growth of cells. This route has been linked to the development of many malignancies, such as TNBC (238). The disruption of the Hippo pathway has been recognized as a possible factor in the emergence and advancement of TNBC. The main mechanism of this route is controlled by a series of kinase reactions that modulate the function of transcriptional coactivator with PDZ-binding motif (TAZ) and Yes-associated protein (YAP) which are essential components of the process (239). When the Hippo pathway is activated, the upstream kinases, MST1/2 and LATS1/2, phosphorylate YAP and TAZ, causing them to be trapped in the plasma membrane and blocking their movement into the nucleus (**Figure 16**). Consequently, the process of gene transcription controlled by YAP/TAZ is suppressed (240).

The Hippo pathway acts as a tumor suppressor by inhibiting the activity of YAP and TAZ, which are known to promote cell proliferation and survival. Dysregulation of the pathway, such as inactivating mutations or altered expression of its components, can result in YAP/TAZ activation, leading to uncontrolled cell growth and tumor formation (246). This Activation has also been associated with the initiation of EMT in TNBC cells, enhancing their capacity to move, infiltrate neighboring tissues, and spread to remote locations. Multiple studies have shown that stimulation of YAP in TNBC cells could have a role in making the cells resistant to chemotherapy. This resistance is believed to occur via the regulation of genes that take part in removing drugs from the cells, repairing DNA damage, and preventing cell death (247).

#### Combination strategies (monoclonal and non- monoclonal antibodies) against hippo pathway

Investigations into monoclonal antibodies that target the Hippo pathway remain in preliminary phases; nonetheless, several techniques are being explored to control this system in TNBC and other malignancies (248). Current Investigations on Monoclonal Antibodies Directed at the Hippo Pathway in TNBC includes:

♣ Monoclonal Antibodies Targeting YAP and TAZ: The development of anti-YAP/TAZ monoclonal antibodies, which directly target YAP/TAZ or block their nuclear activity, remains in the preliminary stages of research. These antibodies are designed to inhibit the interaction between YAP/TAZ and TEAD transcription factors, which facilitates the production of genes that promote cancer (249). Directly targeting YAP/TAZ with monoclonal antibodies is challenging owing to the intricate molecular relationships these proteins maintain with many biological components (246).

♣ TEAD Inhibitors and Monoclonal Antibodies: The connection between TEAD and YAP/TAZ is a primary therapeutic target. Monoclonal antibodies that inhibit the interaction between YAP/TAZ and TEAD are currently being studied. Verteporfin (a YAP-TEAD inhibitor) a tiny compound have shown some effectiveness in preclinical animals and are now advancing to clinical trials for TNBC. Researchers are now investigating the potential of engineered mAbs to disrupt this interaction with greater specificity and potency (244).

♣ Targeting MST1/2 Kinases: MST1 and MST2 serve as upstream regulators of the Hippo pathway, facilitating the phosphorylation and activation of LATS1/2 kinases, which then phosphorylate and inactivate YAP/TAZ. Activation of MST1/2 kinase reinstates the Hippo pathway and diminishes YAP/TAZ activity (241). Targeting MST1/2 may restore normal Hippo signaling in TNBC, thus inhibiting tumor growth and invasion. Although small compounds aimed at MST1/2 are being explored in preclinical investigations, mAbs for MST1/2 activation are not yet broadly accessible. The emphasis is on small chemical activators of MST1/2, which may subsequently facilitate the creation of specific antibodies (250). Researchers are exploring combination treatments including immune checkpoint inhibitors or chemotherapy to enhance the effectiveness of Hippo pathway inhibitors. They are also evaluating mAbs that target the Hippo pathway in conjunction with chemotherapy or immune checkpoint inhibitors (e.g., anti-PD-1/PD-L1, anti-CTLA-4) to improve the treatment response in TNBC. Combination therapy that simultaneously target YAP/TAZ activity and immune suppression may provide synergistic results for TNBC patients, who are often resistant to conventional treatments (242).

♣ mAbs aimed at the Hippo pathway in TNBC remain in preliminary development, with current research concentrating on YAP/TAZ inhibitors, TEAD inhibitors, and MST1/2 activators (239). Presently, there is a significant emphasis on using small compounds that target this route; however, monoclonal antibodies are being developed to possibly provide a more precise and enduring treatment (251).

## Conclusion

TNBC exhibits the poorest prognosis of any kind of breast carcinoma in women. Many characteristics of TNBC contribute to its exceptional aggressiveness compared to other varieties of breast carcinoma. Therefore, comprehending the molecular signaling mechanism of TNBC in the tumor microenvironmnt by the implementation of interdisciplinary research would significantly enhance the diagnosis and treatment of TNBC. An extensive understanding of the genetic and proteomic mechanisms associated with TNBC may aid in the development of innovative treatment approaches and the effective design of clinical trials. The unfavorable clinical result of TNBC treatment may be attributed to cancer recurrence, metastasis, and death. Exploring several signaling pathways in depth and identifying the molecular targets for TNBC therapy is a valuable endeavour. Advancements in computer biology, together with breakthroughs in immunology, nanotechnology, and molecular biology, will provide early detection and customised therapy. Multiple studies are underway, and new targets are being identified, but they have yet to be successfully implemented. A Comprehensive table summarizing the key components of each signaling pathway and the corresponding potential therapeutic strategies is provided in **Table 1**. Scientists are motivated to continually investigate different signaling cascades to discover effective medications with a high rate of pathological complete response (pCR) for the treatment of TNBC. Presently, clinical trials are underway to assess the safety and effectiveness of several medications that target various routes in combination for patients with TNBC. The outcomes of these studies are currently pending.

**Table 1:** Comprehensive table summarizing the key components of each signaling pathway and the corresponding potential therapeutic strategies.

Signaling pathway	Key components	Therapeutic strategies	Monoclonal antibody (MA)	Clinical/preclinical status	NCT no	Direct/indirect effect	References
PI3K Activation	RTKs receptor activation	PI3K antagonists in combination with MA	Herceptin & Margetuximab	CT	NCT00045032	Indirect (by inhibiting HER2)	(33)
				PCT	NA	Indirect (by inhibiting HER2)	(34)
			Anti-PD-L1 antibodies (Atezolizumab, Durvalumab)	CT	NCT02008227, NCT03519971	Indirect (stimulate T cells)	(35)
			Bavituximab	CT	NCT00129337	Indirect (stimulate PI3K/Akt)	(37)
			CureD3	PCT	Not found	Indirect (Stimulate PI3K)	(29)
AKT (Protein Kinase B) Activation	PI3K pathway overactivation	AKT inhibitor in combination with other cancer therapies	Atezolizumab and Durvalumab	CT	NCT03908814	Indirect (stimulate Akt)	(46)
			Antibodies targeting the Anti-Insulin-Like Growth Factor Receptor (IGF-1R) figitumumab	CT	NCT03498417	Indirectly influence Akt	(48)
			Anti-VEGF (Bevacizumab)	CT	NCT02634333	indirectly influence Akt activation	(50)
			Antibodies targeting Interleukin-6 (IL-6) Tocilizumab	CT	NCT04320615	indirectly modulate Akt signaling (by blocking IL-6 receptor signaling)	(55)
mTOR (Mammalian Target of Rapamycin) Activation	Stimulation of beneficial regulators like AKT and PI3K	mTOR can be targeted by direct inhibitors, upstream PI3K/AKT blockers, AMPK activators (e.g., metformin), dietary amino acid restriction, and combination cancer therapies.	Anti-PD-L1 Antibodies (e.g., Atezolizumab, Durvalumab)	CT	NCT02431208, NCT03519971	Indirect effect	(69)
			Anti-IGF-1R antibodies	CT	NCT06866548	Indirect effect	(71)
			Anti-VEGF antibodies	CT	NCT03503604	Indirect effect	(72)
NF-κB (Nuclear Factor-κB) Activation	Canonical NF-κB pathway activation	Indirectly influence NF-κb signaling in cancer cells and the tumor microenvironment	CD40 Agonistic Monoclonal Antibodies	CT	NCT02482168	Indirectly stimulate NF-κB activation	(82)
			CD137 (4-1BB) agonistic antibodies	PCT	Not Found	indirectly activate NF-κB	(83)
			TLR (Toll-Like Receptor) Agonists	PCT	Not Found	Indirect effect	(87)
			Anti-DR5 (Death Receptor 5) Monoclonal Antibodies	PCT	Not Found	Direct effect	(89, 90)
			Targeting NF-κB Pathway Regulators (Inhibition of IκB Kinases and TAK1)	PCT		Indirect effect	(91)
Wnt/β-Catenin Pathway	PI3K/AKT pathway can activate the Wnt/β-catenin pathway	The Wnt/β-Catenin pathway is often linked to cancer growth, metastasis, and chemoresistance	Anti-LRP5/6 (Low-Density Lipoprotein Receptor-Related Protein 5/6) antibodies	PCT		Direct effect (activate Wnt/β-catenin signaling)	(105)
EGFR (Epidermal Growth Factor Receptor) Pathway	Primarily triggered by the binding of epidermal growth factor (EGF)	It can activate pro-survival pathways that counteract the effects of chemotherapy drugs	AMG 595	CT	NCT01475006	indirectly activate EGFR	(114)
			Combination therapies using EGFR-targeting monoclonal antibodies, such as Cetuximab or Panitumumab	CT	NCT00154102, NCT00364013	indirectly activate EGFR	(116)
			Anti-EGFRvIII Antibodies (Mutant EGFR Variant)	PCT		Direct Effect	(117)
JAK/STAT Cascade.	It can regulate immunological responses Activation of STAT3, stimulates the production of immunosuppressive substances	JAK inhibitors, STAT3 inhibitors, cytokine-blocking antibodies, immune checkpoint inhibitors, and combination therapies.	Anti-IL-6 Receptor Antibodies	CT	NCT04322773	indirectly activate immunological responses	(123)
			Siltuximab	CT	NCT01484275	Indirect Method	(125)
			Anti-GM-CSF monoclonal antibodies	CT	NCT04341116	Indirect Method	(127)
			Anti-CD40 Monoclonal Antibodies	CT	NCT03781414	Indirect Method	(128)
			Directly Targeting JAK1/2 with Monoclonal Antibodies	CT	NCT04644211, NCT06517875	Direct Method	(131)
			Monoclonal Antibodies Targeting IL-4 and IL-13 (Dupilumab)	CT	NCT03346434	Indirect (inhibits IL-4 and IL-13 signaling)	(132, 133)
Notch Pathway:	Activated by a sequence of proteolytic cleavages	Monoclonal antibodies against Notch receptors/ligands, and combination therapies targeting interacting pathways.	Anti-Jagged1 Monoclonal Antibodies (UHD 156)	PCT	Not Found	Direct method	(147)
			Anti-Delta-like Ligands (DLL) Monoclonal Antibodies (PF-06747775)	CT	NCT02349633	Direct method	(149)
			Anti-Notch3 mAbs	PCT		Direct method	(150)
			Monoclonal antibodies directed against the Notch intracellular domain (NICD)			Direct method	(152)
			Notch Agonistic Antibodies	PCT		Direct method	(154)
DNA Damage Response (DDR) Pathway:	BRCA1 is an important DDR gene	This involves targeting essential DDR proteins or augmenting DDR activity in tumor cells to provoke cell death or increase tumor sensitivity to radiation or chemotherapy	Anti-ATM Monoclonal Antibodies	CT	NCT06775236	Indirect method	(166)
			Anti-ATR Monoclonal Antibodies	CT	NCT05338346	Indirect method	(168, 170)
			Anti-PARP monoclonal antibodies	CT	NCT04681469	Indirectly target the PARP enzyme	(171)
			Anti-BRCA Monoclonal Antibodies (Targeting DNA Repair Pathways)	PCT		Indirectly (target BRCA1/2 proteins)	(172)
			Anti-Checkpoint Kinase 1 (CHK1) Antibodies	PCT		Indirect method (target CHK1 kinase indirectly)	(173)
Hedgehog Pathway:	Activation of cancer stem cells (cscs)	By improving the production of drug efflux pumps, like ATP-binding cassette transporters	Vismodegib (GDC-0449)	CT	NCT00636610	Direct Method	(187)
			Sonidegib (LDE225)	CT	NCT02027376	Direct inhibitor of SMO	(188)
			Anti-Patched1 (PTCH1) Antibodies	PCT		Directly (by binding to the PTCH1 receptor)	(189)
			Anti-GLI Transcription Factor Antibodies	PCT		Directly by targeting GLI1 and GLI2	(190)
			Anti-Hedgehog Ligand (Shh) Antibodies	PCT		Directly (by binding to the Shh ligand)	(186)
MET Pathway	It is activated by the hepatocyte growth factor (HGF)	MET inhibitors, anti-MET and anti-HGF antibodies, and combination therapies.	Inhibitory Anti-MET Monoclonal Antibodies (such as onartuzumab, savolitinib)	CT	NCT01456325, NCT03091192	Directly block the MET receptor	(198)
			Onartuzumab (MetMAb)	CT	NCT01456325	Direct method	(196)
			Savolitinib (AZD6094)	CT	NCT03091192	Direct method	(199)
			Anti-HGF Monoclonal Antibodies	PCT		Direct method (binding to HGF)	(205)
HER2 signaling (Amplification)	MAPK and PI3K/Akt pathways	Therapeutic strategies targeting HER2 in TNBC include monoclonal antibodies, tyrosine kinase inhibitors, antibody-drug conjugates, and combination therapies.	Margetuximab (MGAH22)	CT	NCT01828021	Direct method	(207)
			Pertuzumab (Perjeta)	CT	NCT02738970	Direct method	(208)
			HER2-Targeted Biologic Therapies with Modulatory Effects.	CT	NCT00021255	Direct method	(210)
			Sacituzumab Govitecan	CT	NCT04468061	Indirect method	(212)
			Bispecific Antibodies: Blinatumomab (Blincyto)	CT	NCT01466179	Direct method	(215)
			Current and Upcoming Clinical Trials in HER2-Targeted Therapy for TNBC (Atezolizumab (anti-PD-L1))	CT	NCT01846416	Direct method	(216)
			The HER2CLIMB study	CT	NCT02614794	Direct method	(224)
TGF-β (Transforming Growth Factor-beta) Pathway	TGF-βRII phosphorylates and promotes TGF-βRI	TGF-β pathway include TGF-β ligand traps, receptor kinase inhibitors, anti-TGF-β monoclonal antibodies, and Smad pathway modulators.	Monoclonal Antibodies Against TGF-β: Fresolimumab (GC1008)	CT	NCT00356460	Directly to TGF-β ligands	(227)
			M7824	CT	NCT02517398	Direct method (blocking TGF-βRII to inhibit TGF-β signaling)	(229)
			Trabedersen (AP 12009)	CT	NCT00431561	Indirectly by targeting TGF-β2 mrna	(231)
FAK (Focal Adhesion Kinase) Pathway	Initiating downstream signaling pathways, such as the PI3K/Akt and MAPK pathways	FAK inhibitors, anti-FAK antibodies, and combination therapies.	Defactinib (VS-6063)	CT	NCT02004028	Direct method	(238)
STAT3 (Signal Transducer and Activator of Transcription 3) Pathway	STAT3 stimulates pro-angiogenic substances, fibroblast growth factor (FGF), interleukin-8 (IL-8) and VEGF	STAT3 inhibitors, blocking antibodies, and combination therapies.	Monoclonal Antibodies Directed Against the STAT3 Pathway in TNBC. N-803 and mAb CXXC5	PCT		Direct method	(241)
			CDDO-Me	PCT		Direct method	(242)
			S3I-201 (STAT3 Inhibitor) Combined with Chemotherapy or Immunotherapy	PCT		Direct method	(243)
Hippo Pathway	The upstream kinases, MST1/2 and LATS1/2, phosphorylate YAP and TAZ	Inhibit YAP/TAZ activity or activate MST/LATS kinases to suppress TNBC growth	Monoclonal Antibodies Targeting YAP and TAZ	CT	NCT04857372	Directly target YAP/TAZ	(244)
			TEAD Inhibitors and Monoclonal Antibodies	CT	NCT05228015	Direct method	(241)
			Targeting MST1/2 Kinases	PCT		Indirect method	(245)

## Glossary

ADC: Antibody-drug conjugate
AKT: Protein Kinase B
APC: Adenomatous polyposis coli
AR: Androgen receptor
CDK4: Cyclin-dependent kinase 4
CK1: Casein kinase 1
CNVs: Copy number variations
COX-2: Cyclooxygenase-2
CSCs: Cancer stem cells
CTLA-4: Cytotoxic T-Lymphocyte-Associated Protein 4
DLL: Delta-like ligands
DSBs: Double-strand breaks
ECM: Extracellular matrix
EGF: Epidermal growth factor
EGFR: Epidermal Growth Factor Receptor
EMT: Epithelial-mesenchymal transition
ER: Estrogen receptor
EffNetb0: EfficientNet-b0
FAK: Focal adhesion kinase
FGF: Fibroblast growth factor
GSK3β: Glycogen Synthase Kinase 3 Beta
HGF: Hepatocyte growth factor
HER2: Human epidermal growth factor receptor 2
HRD: Homologous Recombination Deficiency
IFN-γ: Interferon-gamma
IGF-1R: Insulin-like Growth Factor 1 Receptor
IKKβ: Inhibitor of nuclear factor kappa-B kinase subunit beta
IL-6: Interleukin-6
IL-8: Interleukin-8
INPP4B: Inositol polyphosphate-4-phosphatase type II
JAK: Janus kinase
LRP: Low-density lipoprotein receptor-related protein
mAb: Monoclonal antibody
MMP-9: Matrix Metalloproteinase-9
MMPs: Metalloproteinases
mTOR: Mammalian Target of Rapamycin
NF-κB: Nuclear factor-κB
NICD: Notch intracellular domain
PAMPs: Pathogen-associated molecular patterns
PD-1: Programmed cell death protein 1
PDK1: Phosphoinositide-dependent protein kinase-1
PD-L1: Programmed death-ligand 1
PI3K: Phosphoinositide 3-kinase
PIK3CA: PI3K catalytic subunit
PIP2: Phosphatidylinositol 4,5-bisphosphate
PIP3: Phosphatidylinositol 3,4,5-trisphosphate
PR: Progesterone receptor
PTEN: Phosphatase and tensin homolog
ResizeMix: ResizeMix augmentation method
RNet18: Residual Network-18
RNet34: Residual Network-34
RNet50: Residual Network-50
RTKs: Receptor tyrosine kinases
SMO: Smoothened
STAT3: Signal Transducer and Activator of Transcription 3
TAK1: Transforming growth factor beta-activated kinase 1
TCF/LEF: T-cell factor/lymphoid promoter factor
TEAD: Transcriptional enhanced associate domian
TGF-β: Transforming growth factor-beta
TEAD: Transcriptional enhanced associate domain
TGF-β: Transforming growth factor-beta
TNBC: Triple negative breast cancer
TRAIL: TNF-related apoptosis-inducing ligand
VEGF: Vascular endothelial growth factor
YAP: Yes-associated protein
